# *Toxoplasma* effector *Tg*WIP hijacks dendritic cell actin and motility via Nck1/Grb2 and the WAVE complex

**DOI:** 10.1128/mbio.01874-25

**Published:** 2025-10-31

**Authors:** Pavel Morales, Daniel A. Kramer, Caroline de Moraes de Siqueira, Abbigale J. Brown, Lamba Omar Sangaré, Baoyu Chen, Jeroen P. J. Saeij

**Affiliations:** 1 Department of Pathology, Microbiology and Immunology, School of Veterinary Medicine, University of California 8789, Davis, California, USA; 2 Roy J. Carver Department of Biochemistry, Biophysics & Molecular Biology, Iowa State University 1177, Ames, Iowa, USA; 3 Departments of Internal Medicine and Biophysics, University of Texas Southwestern Medical Center 12334, Dallas, Texas, USA; University of Wisconsin-Madison, Madison, Wisconsin, USA

**Keywords:** *Toxoplasma gondii*, *Tg*WIP effector, dendritic cell motility, Nck1, Grb2, WAVE regulatory complex, proline-rich region, WIRS motif, podosome, cell motility, actin cytoskeleton remodeling

## Abstract

The intracellular parasite *Toxoplasma gondii* enhances its dissemination to distant organs by hijacking infected leukocytes via a Trojan Horse mechanism. Upon infecting dendritic cells (DCs), *Toxoplasma* induces a hypermigratory phenotype characterized by podosome dissolution and formation of F-actin stress fibers. We previously showed that these cytoskeletal changes depend on the effector protein *Toxoplasma* WAVE complex-interacting protein (*Tg*WIP) secreted from parasites to infected leukocytes. Here, we identify the host adaptor proteins non-catalytic region of tyrosine kinase adaptor protein 1 and 2 (Nck1/2) and growth factor receptor-bound protein 2 (Grb2) as direct *Tg*WIP interactors. *Tg*WIP mainly uses two distinct proline-rich regions (PRRs) to interact with Nck1 and Grb2. Mutating these PRRs abrogates *Tg*WIP binding to Nck1 and Grb2 and diminishes podosome dissolution and DC hypermotility. Furthermore, we show that *Tg*WIP directly interacts with the actin nucleation-promoting factor WAVE regulatory complex (WRC) via a WRC-interacting receptor sequence (WIRS). Disrupting this interaction also influences actin cytoskeletal remodeling and DC hypermotility. Collectively, our data reveal that *Tg*WIP directly interacts with multiple actin regulators, including Nck1, Grb2, and the WRC, to remodel the actin cytoskeleton of the host cells, elucidating a key mechanism that *Toxoplasma* exploits to enhance host cell migration and dissemination.

**IMPORTANCE:**

The parasite *Toxoplasma gondii* spreads throughout the body by hijacking immune cells and boosting their motility. This ability depends on secreted parasite proteins that manipulate the host cell’s actin cytoskeleton. One such effector, *Toxoplasma gondii* WAVE-interacting protein (*Tg*WIP), induces dramatic changes in host cell shape and movement, but how it does this has remained unclear. Here, we show that *Tg*WIP directly interacts with multiple host actin-regulatory proteins using distinct sequence motifs. Disrupting these interactions prevents cytoskeletal remodeling and impairs parasite-induced immune cell migration. Our study reveals that *Toxoplasma* uses defined motifs to co-opt host signaling hubs that control cell motility. Understanding how pathogens exploit the cytoskeleton not only sheds light on host-pathogen interactions but may also reveal broader principles of cell migration relevant to immunity, cancer, and development.

## INTRODUCTION

The actin cytoskeleton is a highly dynamic network that regulates crucial cellular functions, including cell shape, motility, and internal trafficking (1). Intracellular pathogens have evolved numerous ways to exploit this network to promote their survival, replication, and dissemination. A common strategy is the secretion of virulence factors that modulate host regulators of actin dynamics (2). Some of these factors use host-mimicry motifs to engage cytoskeletal regulators, inducing actin rearrangements to dampen immune responses or facilitate pathogen spread within and between host cells (3–5). Identifying the molecular mechanisms by which microbial effectors hijack the host cytoskeleton can provide insights into how pathogens manipulate host cells during infection.

*Toxoplasma gondii* is an obligate intracellular pathogen capable of infecting a wide range of warm-blooded vertebrates, including humans and rodents. After oral infection, *Toxoplasma* disseminates broadly in the organism to reach peripheral organs, including the central nervous system (6). While chronic infection is mainly asymptomatic, acute or reactivated infection can cause life-threatening disease in the developing fetus and in immunocompromised individuals (7, 8).

*Toxoplasma* actively invades peripheral tissues by hijacking leukocytes, such as macrophages and dendritic cells (DCs) (9, 10). Infected DCs rapidly undergo a hypermigratory phenotype characterized by cytoskeletal rearrangements and dramatically enhanced motility (hypermotility) (11). *Toxoplasma* induces similar hypermotility features in otherwise immobile macrophages, potentiating parasite dissemination (12). Infected DCs undergo dissolution of podosomes—actin-rich adhesive structures that degrade the extracellular matrix via matrix metalloproteinase secretion—and assemble F-actin stress fibers that promote contractility and motility (13–15). These cytoskeletal changes have been linked to enhanced systemic dissemination in mice via a Trojan Horse mechanism, whereby infected leukocytes serve as vehicles for parasite transport and immune escape (16–18).

Although the molecular pathways underlying *Toxoplasma*-induced DC hypermotility remain incompletely defined, they clearly depend on secreted parasite effectors. For example, the *Tg*14-3-3 effector induces hypermotility in both DCs and microglia (19), while the secreted protein kinase ROP17 enhances monocyte locomotion on endothelial surfaces (20). Another key effector is the *Toxoplasma gondii* WAVE-interacting protein (*Tg*WIP), which enhances leukocyte motility independently of toll-like receptor signaling and chemotactic cues (11, 13, 14). *Tg*WIP is a rhoptry protein secreted into the host cytosol, where it drives podosome dissolution and the hypermotility phenotype of infected DCs (13, 14). Mass spectrometry analysis revealed that *Tg*WIP associates with multiple host actin regulators, including Src-homology 2 domain (SH2)-containing phosphatase 1 and 2 (Shp1/2), non-catalytic region of tyrosine kinase adaptor protein 1 and 2 (Nck1/2), growth factor receptor-bound protein 2 (Grb2), and components of the WAVE regulatory complex (WRC) (13). We previously showed that *Tg*WIP is phosphorylated by host Src kinases at Y150 and Y199, which subsequently bind to and activate Shp1/2 (14). Whether *Tg*WIP directly interacts with Nck1/2 or WRC remains unknown.

Nck1 and Grb2 are key SH2/SH3 adaptor proteins that couple factors containing phosphorylated tyrosines via their SH2 domains or proline-rich regions (PRRs) via their SH3 domains to actin remodeling pathways (21, 22). Through these interactions, they modulate the activity of Wiskott-Aldrich syndrome (WAS) family proteins, including WASP and N-WASP, to promote Arp2/3-mediated actin polymerization essential for podosome formation and cell adhesion (23–26). Several pathogens, including enteropathogenic *Escherichia coli* and vaccinia virus, hijack Nck1- and Grb2-mediated actin remodeling to promote infection and spread (5, 27, 28). WAVE proteins (WAVE1-3) also drive Arp2/3-dependent actin polymerization essential for cell adhesion and migration (29, 30). WAVE functions within the heteropentameric WRC, which contains four other subunits: Sra1, Nap1, Abi2, and HSPC300 (or their corresponding paralog proteins) (31, 32). Various transmembrane receptors recruit the WRC using a conserved WRC-interacting receptor sequence (WIRS) motif that binds to a conserved surface pocket formed by Sra1 and Abi2 (32, 33).

We previously showed that *Tg*WIP-Shp1/2 interactions drive F-actin stress fiber-mediated hypermotility in DCs (14). However, podosome dissolution occurs independently of this Shp1/2 pathway, suggesting additional mechanisms are involved. Here, we demonstrate that *Tg*WIP’s PRRs directly bind to Nck1 and Grb2, while its WIRS motif binds to the WRC. DCs infected with *Toxoplasma* expressing *Tg*WIP mutants lacking PRR or WIRS sequences fail to exhibit cytoskeletal remodeling or hypermotility. These results reveal a previously unrecognized mechanism through which *Tg*WIP remodels the DC actin cytoskeleton.

## RESULTS

### *Tg*WIP uses distinct PRRs to bind to Nck1 and Grb2

**Fig 1:**
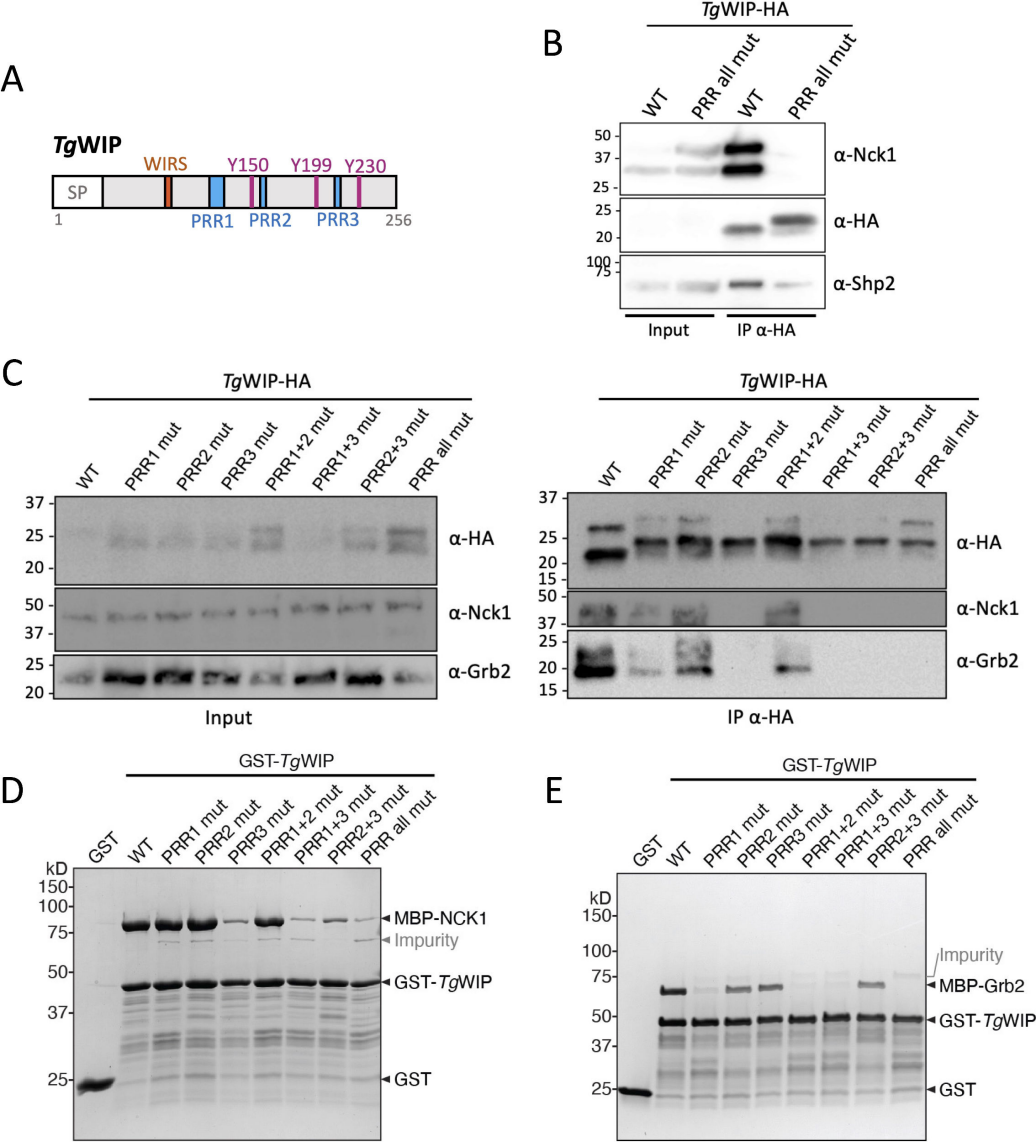
*Tg*WIP uses PRRs to interact with Nck1 and Grb2. (**A**) Schematic showing the amino acid sequence of *Tg*WIP. SP, signal peptide critical for secretion of *Tg*WIP, is excluded from the recombinant protein sequence. (**B**) The murine DC (DC2.4) cell line was infected for 4 h with ME49 ∆*tgwip* parasites complemented with HA-tagged *Tg*WIP^WT^ or *Tg*WIP^PRR all mut^. Total cell lysates (input; ~5%-10% of total lysate) and anti-HA immunoprecipitates (IP α-HA) were sequentially immunoblotted with antibodies to Nck1 and HA after stripping the membrane between probes. (**C**) DC2.4 cells were infected as in panel **B** using ME49 ∆*tgwip* parasites complemented with the indicated *Tg*WIP variants. Total cell lysates (input; ~5%-10% of total lysate) and anti-HA immunoprecipitates (IP α-HA) were blotted and reprobed for Nck1, HA, and Grb2, as in panel **B**. (**D–E**) Coomassie blue-stained SDS-PAGE gels showing various GST-tagged *Tg*WIP constructs pulling down MBP-tagged full-length Nck1 in panel **D** and Grb2 in panel E. Each reaction contained 200 pmol of bait and 1,000 pmol of prey in a pull-down buffer containing 50 or 100 mM NaCl. Representative gels of two independent experiments are shown.

Although *Tg*WIP-Shp1/2 interactions promote the hypermotility phenotype, they do not explain podosome dissolution, suggesting the presence of independent mechanisms. In previous proteomic analysis, Nck1 and Nck2 (Nck1/2) and Grb2 were shown to be potential *Tg*WIP interactors (13). Given the importance of Nck1/2 and Grb2 in actin regulation and podosome formation, we hypothesized that *Tg*WIP uses its PRRs to interact with the SH3 domains of Nck1/2 and Grb2 (Fig. 1A).

To test whether *Tg*WIP’s PRRs bind to Nck1, we infected murine DCs (cell line DC2.4) with *Tg*WIP knockout parasites (∆*tgwip*) complemented with either the wild-type *Tg*WIP with a C-terminal HA tag (*Tg*WIP^WT^) or a mutant in which all three PRRs are substituted with alanine (*Tg*WIP^PRR all mut^, Fig. S1). Subsequently, we immunoprecipitated HA-tagged *Tg*WIP^WT^ vs *Tg*WIP^PRR all mut^ from infected DCs and used Western blot to detect co-immunoprecipitated Nck1 and Shp2 (Fig. 1B). Our results demonstrate that *Tg*WIP^WT^ had robust binding to both Nck1 and Shp2, while *Tg*WIP^PRR all mut^ showed a complete loss of Nck1 binding but retained partial binding to Shp2. This suggests that *Tg*WIP’s PRRs are essential for binding to Nck1, whereas Shp2 binding is only partially reduced and likely mediated mostly through PRR-independent interactions, such as the known phosphotyrosine motifs (Y150 and Y199). This is consistent with prior findings that Shp2 recruitment depends on tyrosine phosphorylation of *Tg*WIP.

To identify which PRRs mediate *Tg*WIP binding to Nck1, we generated ∆*tgwip* parasites complemented with *Tg*WIP variants in which different regions of PRRs are substituted with alanine, including PRR1 (*Tg*WIP^PRR1 mut^), PRR2 (*Tg*WIP^PRR2 mut^), PRR3 (*Tg*WIP^PRR3 mut^), or their pairwise combinations (*Tg*WIP^PRR1+2 mut^, *Tg*WIP^PRR1+3 mut^, *Tg*WIP^PRR2+3 mut^). Immunofluorescence confirmed that, like *Tg*WIP^WT^, all mutants localized to rhoptries (Fig. S2) and were secreted into the host cytosol at levels comparable to *Tg*WIP^WT^ (Fig. S3). HA-coimmunoprecipitation (co-IP) and immunoblotting from infected DCs showed that *Tg*WIP^WT^ and *Tg*WIP^PRR2 mut^ retained detectable binding to Nck1 and Grb2. In contrast, *Tg*WIP^PRR3 mut^, *Tg*WIP^PRR1+3 mut^, *Tg*WIP^PRR2+3 mut^, and *Tg*WIP^PRR all mut^ showed a complete loss of interaction with Nck1 and Grb2, indicating that *Tg*WIP’s PRR3 is crucial for binding to both proteins (Fig. 1C). Mutating PRR1 (*Tg*WIP^PRR1 mut^ and *Tg*WIP^PRR1+2 mut^) led to a reduction, but not complete loss of binding to both Nck1 and Grb2, suggesting that PRR1 contributes to, but is not solely responsible for these interactions.

To corroborate the co-IP data and determine whether *Tg*WIP directly binds to Nck1 and Grb2, we performed *in vitro* GST pull-down assays using purified recombinant proteins. Consistent with the co-IP data, we found that GST-tagged *Tg*WIP^WT^ robustly pulled down MBP-tagged full-length human Nck1 and Grb2, whereas the *Tg*WIP^PRR all mut^ had greatly reduced binding to either protein, confirming that the interactions are PRR-dependent (Fig. 1D and E). Consistent with the co-IP results, *Tg*WIP^PRR3 mut^ and the PRR3-containing double mutants (*Tg*WIP^PRR1+3 mut^ and *Tg*WIP^PRR2+3 mut^) failed to bind Nck1, identifying PRR3 as essential for this interaction. In contrast, *Tg*WIP^PRR1 mut^ retained Nck1 binding, indicating that PRR1 is dispensable for direct Nck1 interaction *in vitro* (Fig. 1D). For Grb2, *Tg*WIP^PRR1 mut^ had a clear loss of binding, while *Tg*WIP^PRR3 mut^ retained binding, indicating that PRR1 is the primary determinant of the direct interaction with Grb2 (Fig. 1E). These results suggest that *Tg*WIP binds Nck1 and Grb2 through distinct PRRs, and that the relative importance of PRR1 and PRR3 differs slightly between cellular and *in vitro* contexts, possibly due to additional host factors or conformational influences present in cells but absent in the reconstituted system. These interactions, confirmed *in vitro*, suggest direct binding.

### *Tg*WIP uses a WIRS motif to bind the WRC

**Fig 2:**
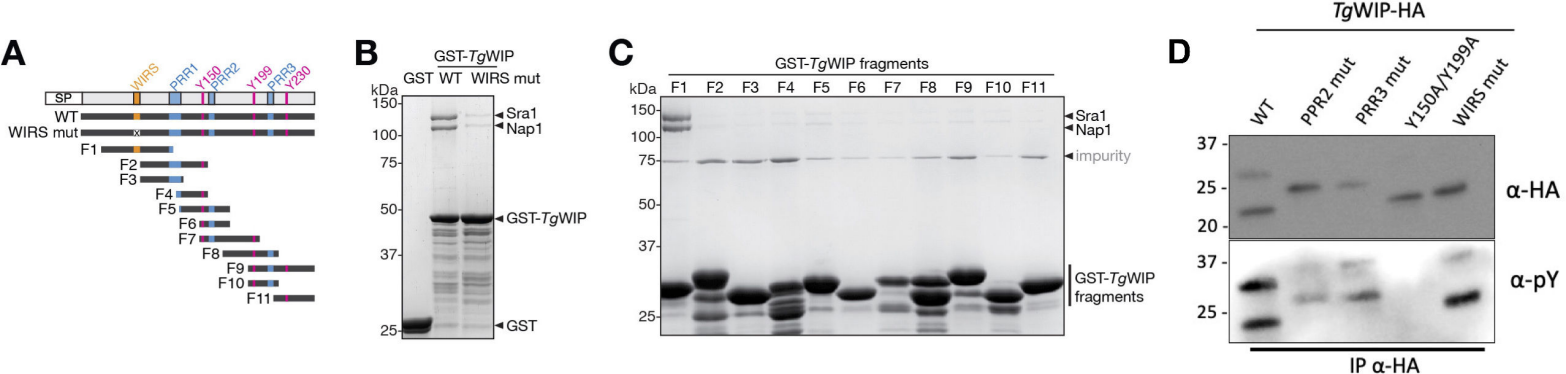
*Tg*WIP engages the WAVE regulatory complex through its WIRS motif while retaining independent Src-mediated tyrosine phosphorylation. (**A**) Schematic showing various purified *Tg*WIP fragments. (**B–C**) Coomassie-blue-stained SDS PAGE gels showing GST-*Tg*WIP pulling down recombinantly purified WRC. Each reaction contained 200 pmol of bait and 300 pmol (**B**) or 60 pmol (**C**) of prey in a pull-down buffer containing 100 mM (**B**) or 50 mM (**C**) NaCl. The bands corresponding to WAVE1, Abi2, and HSPC300 subunits of WRC were out of the gel separation range due to their low molecular weights. Representative gels of two independent experiments are shown. (**D**) DC2.4 cells were infected with *Tg*WIP^WT^, *Tg*WIP^PRR2 mut^, *Tg*WIP^PRR3 mut^, *Tg*WIP^Y150A/Y199A^, or *Tg*WIP^WIRS mut^ parasites and immunoprecipitated. Shown are the Western blots using HA (first membrane-probing antibody) and phosphotyrosine (pY) (second membrane-probing antibody after stripping membrane) antibodies on total lysate or immunoprecipitated *Tg*WIP.

Previous mass spectrometry data identified all subunits of the WRC as *Tg*WIP binding partners (13). *Tg*WIP contains a WIRS motif (FGTFVK, corresponding to ɸ-x-T/S-F-x-x, where ɸ represents bulky hydrophobic residues and x for any amino acid) (Fig. 1A). Therefore, we hypothesized that this motif mediates the interaction between *Tg*WIP and WRC. Although WRC subunits are difficult to detect by conventional Western blot following co-IP, interaction with the WRC has been consistently observed by mass spectrometry. To determine whether *Tg*WIP binds the WRC directly, we performed pull-down assays using purified, fully assembled recombinant WRC, following protocols used in the original identification of the WIRS motif in transmembrane proteins (32). We found that GST-*Tg*WIP, but not GST alone, robustly bound to the WRC in pull-down assays (Fig. 2B). Mutating the WIRS motif with alanines substantially reduced the binding (Fig. 2B). In parallel, we divided the *Tg*WIP sequence into 11 fragments and purified each fragment as a GST-tagged protein (Fig. 2A and C). Among them, only the F1 fragment, which contains the WIRS motif, was able to pull down the WRC (Fig. 2C). Together, these results confirm that *Tg*WIP directly interacts with the WRC via its WIRS motif. This suggests that while the WIRS motif is necessary for direct binding, additional contacts involving *Tg*WIP’s PRRs, such as with SH3 domains in WRC subunits or associated adaptors, may contribute to WRC recruitment in the cellular context.

### *Tg*WIP interacts with Nck1/Grb2 and WRC independently of Shp1/2 interaction

Previous studies established that two tyrosine residues (Y150 and Y199) in *Tg*WIP become phosphorylated by host Src-family kinases (14). Once phosphorylated, these sites recruit and activate the host tyrosine phosphatases Shp1 and Shp2, subsequently altering the DC actin cytoskeleton and motility. To test whether mutations in *Tg*WIP’s PRRs or WIRS motif affect its phosphorylation status, we immunoprecipitated *Tg*WIP^WT^, *Tg*WIP^PRR2 mut^, *Tg*WIP^PRR3 mut^, *Tg*WIP^Y150A/Y199A^, and *Tg*WIP^WIRS mut^ from infected DCs and probed each with anti-phosphotyrosine antibodies. As expected, the Y150A/Y199A double mutant lacked phosphotyrosine signal, confirming that these residues are the primary Src target. All other variants showed detectable, although somewhat less intense, phosphotyrosine signals, compared to *Tg*WIP^WT^ (Fig. 2D). Together, these findings suggest that mutating individual PRRs or the WIRS motif does not abolish Src-dependent *Tg*WIP phosphorylation. This supports the idea that *Tg*WIP engages Shp1/2, Nck1/Grb2, and the WAVE complex, via distinct structural motifs.

### Mutations in *Tg*WIP impair its ability to induce actin cytoskeletal rearrangements

**Fig 3:**
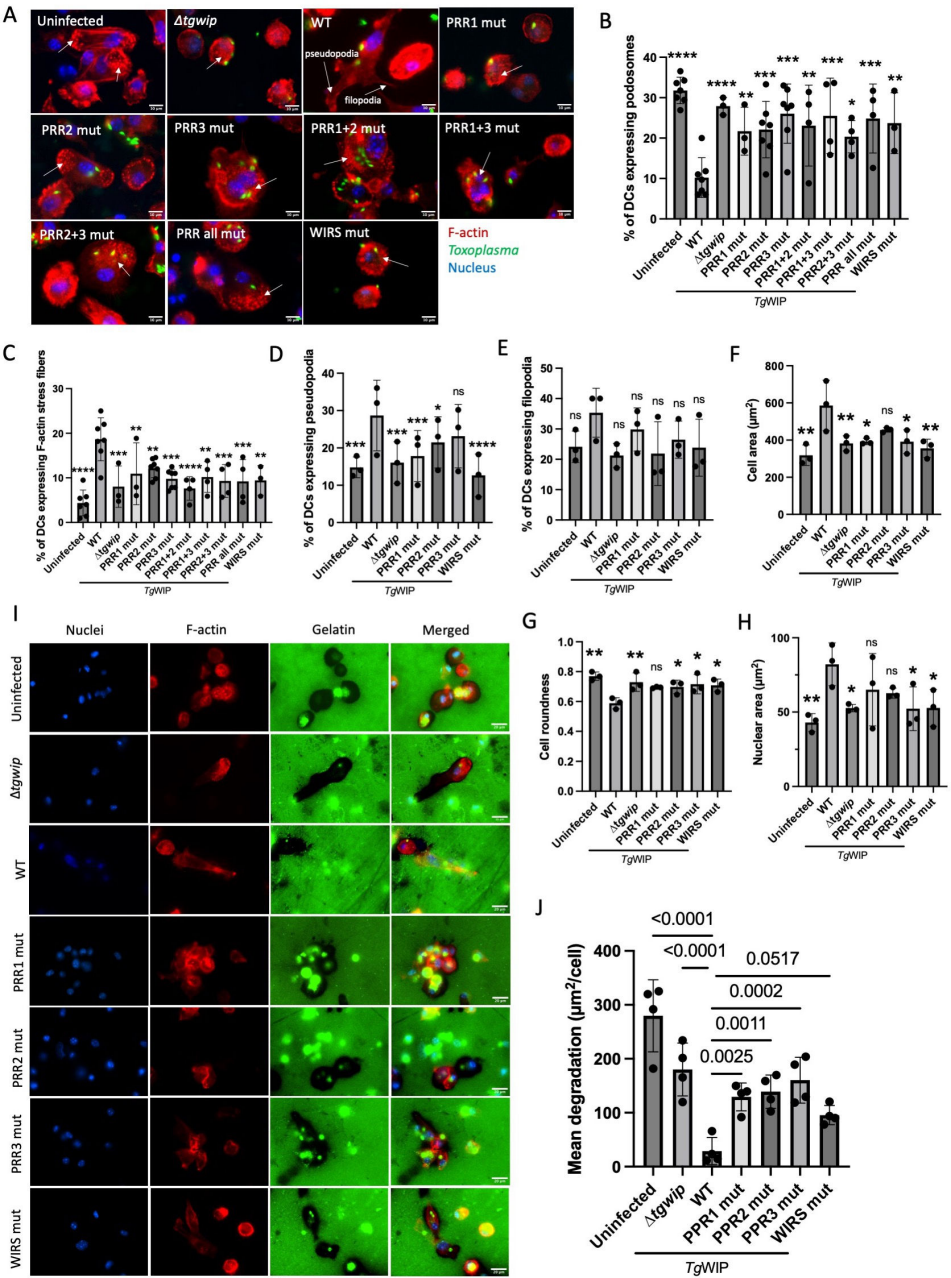
Proline-rich regions and WIRS motif of *Tg*WIP control actin remodeling and matrix degradation in BMDCs. (**A–H**) BMDCs were left uninfected or infected for 4 h with the indicated *Toxoplasma* strains. F-actin and podosomes were visualized with 594 Alexa Fluor Phalloidin, parasites with expressed GFP, and nuclei with DAPI. Unless otherwise indicated, white arrows indicate podosome-expressing DCs. Quantifications are shown at 4 h post-infection of the percentage of BMDCs containing (**B**) podosomes, (**C**) F-actin stress fibers, (**D**) pseudopodia, (**E**) filopodia, (**F**) cell roundness, (**G**) cell area, and (**H**) nuclear area. (**I**) Representative images of pericellular gelatin degradation of BMDCs infected with the indicated parasite strains. Loss of fluorescence from FITC-gelatin marks proteolyzed zones. Bright green puncta within the gelatin represent GFP-expressing parasites. F-actin is shown with phalloidin. (**J**) Mean gelatin degradation area per field of view normalized to the cell number (μm^2^/cell) for DCs infected with the indicated parasites. Data are from at least three independent experiments. Significance was determined by two-way analysis of variance (ANOVA), followed by Dunnett’s multiple comparisons. Symbols (*), (**), (***), and (****) indicate statistically significant differences compared to *Tg*WIP^WT^, with *P*-values of <0.05, <0.005, <0.0002, and <0.0001, respectively; ns, not significant.

We previously showed that *Tg*WIP’s interactions with the tyrosine phosphatases Shp1 and Shp2 via its SH2-binding motifs Y150 and Y199 are required for F-actin stress-fiber formation in bone-marrow-derived dendritic cells (BMDCs) (14). However, these interactions did not contribute to *Tg*WIP-mediated dissolution of podosomes, indicating that *Tg*WIP dissolves podosomes in BMDCs via interactions with other host proteins. To test whether the interactions with Nck1/Grb2 or WRC contribute to podosome dissolution, we infected BMDCs with ∆*tgwip* parasites complemented with either *Tg*WIP^WT^ or individual PRR or WIRS mutants (Fig. 3A). Consistent with a previous report (14), BMDCs infected with *Tg*WIP^WT^ parasites exhibited dramatic cytoskeletal and morphological alterations compared to uninfected or ∆*tgwip-*infected cells, exhibiting increased F-actin stress fibers, membrane protrusions, cell area, and nuclear area, and reduced cell roundness (Fig. 3F through H). In contrast, DCs infected with the *Tg*WIP^PRR3 mut^ or *Tg*WIP^PRR1 mut^ strains showed podosome dissolution and F-actin stress-fiber levels similar to ∆*tgwip* parasites. To our surprise, *Tg*WIP^PRR2 mut^ also exhibited similar phenotypes, even though PRR2 was apparently not important for the interaction with Nck1 or Grb2 (Fig. 1), suggesting that PRR2 may mediate interactions with additional SH3-containing host proteins involved in actin remodeling. Similarly, BMDCs infected with *Tg*WIP^WIRS mut^ parasites exhibited increased podosome expression, reduced F-actin stress fiber levels, fewer membrane protrusions, decreased cellular and nuclear area, and greater cell roundness compared to *Tg*WIP^WT^ infected BMDCs (Fig. 3A through H). These results indicate that *Tg*WIP’s PRRs and WIRS motif play a role in modulating the actin cytoskeleton of DCs via interactions with Nck1, Grb2, WRC, and likely other host proteins (Fig. 3A and B).

To further investigate the role of *Tg*WIP’s PRRs and WIRS in dysregulating podosome formation in DCs, we quantified gelatin matrix degradation as a functional readout of podosome activity, a widely used assay that assesses podosome function by quantifying matrix degradation (15). DCs infected with ∆*tgwip*, *Tg*WIP^PRR1 mut^, *Tg*WIP^PRR2 mut^, *Tg*WIP^PRR3 mut^, and *Tg*WIP^WIRS mut^ parasites (Fig. 3I) displayed significantly greater gelatin degradation than those infected with *Tg*WIP^WT^ parasites (Fig. 3J), indicating impaired podosome dissolution. These results show that both the PRRs and the WIRS motif are individually required for efficient suppression of podosome function and reorganization of the actin cytoskeleton in infected DCs, most likely through interactions with Nck1/2, Grb2, and associated effectors.

### *Tg*WIP mutants could not promote hypermotility

**Fig 4:**
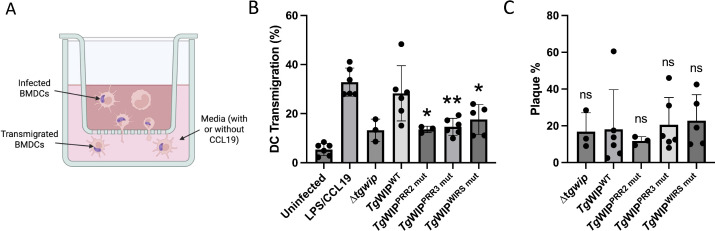
Transwell migration and plaque assays reveal that *Tg*WIP PRRs and WIRS motif promote DC hypermotility. (**A**) Experimental design for the transwell transmigration assay. BMDCs were left uninfected or infected for 4 h with indicated parasites, then loaded in the upper chamber of the transwell. After an 18 h incubation, the cells that migrated to the lower chamber were counted. As a positive control, uninfected BMDCs stimulated for 24 h with LPS were allowed to migrate toward the chemokine CCL19. (**B**) Transmigration frequency for each condition is expressed as the percentage of total cells added to the insert. Bars show means ± SEM from at least three independent experiments, each performed in duplicate. (**C**) Plaque assay assessing the viability of each parasite strain. Bars show mean plaque numbers from the same independent experiments as in panel **B**, each performed in duplicate. Statistics were calculated with two-way ANOVA, followed by a Dunnett’s multiple comparisons test. Symbols (*), (**), and (****) indicate statistically significant differences compared to *Tg*WIP^WT^, with *P*-values of <0.05, <0.005, and <0.0001, respectively; ns, not significant.

Previous studies have shown that DCs infected with *Toxoplasma* have increased migration across a transwell containing a porous membrane, and this hypermotility phenotype is dependent on *Tg*WIP (11, 13). To test the role of *Tg*WIP PRRs and WIRS motif in modulating the migration of DCs, we performed transwell transmigration assays comparing DCs infected with *Tg*WIP^WT^, *Tg*WIP^WIRS mut^, and *Tg*WIP^PRR mut^ strains. Uninfected DCs displayed low levels of transmigration, while priming the cells with lipopolysaccharide (LPS) and adding the DC chemoattractant CCL19 to the bottom chamber of the transwell significantly increased transmigration (Fig. 4A and B). Similar to LPS/CCL19 treatment, infecting DCs with *Tg*WIP^WT^ parasites also significantly increased transmigration, consistent with previous data. In contrast, DCs infected with *Tg*WIP^PRR2 mut^, *Tg*WIP^PRR3 mut^, *Tg*WIP^WIRS mut^, or ∆*tgwip* parasites displayed significantly lower transmigration compared to *Tg*WIP^WT^ infected DCs. The reduced transmigration was not due to loss of parasites, because the mutations did not affect parasite viability in our plaque assays (Fig. 4B and C). Together, our data suggest that *Tg*WIP’s PRRs and WIRS motif are required for elevated transmigration rates in DCs.

### Mass spectrometry analysis reveals additional *Tg*WIP ligands involved in actin cytoskeletal regulation

Our western blot and biochemical analyses show that *Tg*WIP binding to Nck1 and Grb2 is mediated chiefly by *Tg*WIP’s PRR3 and PRR1, but not PRR2 (Fig. 1). However, DCs infected with *Tg*WIP^PRR2 mut^ also induced actin cytoskeletal rearrangements and low transmigration rates similar to DCs infected with *Tg*WIP^PRR3^ and *∆tgwip* (Fig. 3 and 4), indicating the involvement of other host proteins that could modulate actin dynamics in DCs through PRR2. To identify such factors and validate the dependence on PRR1, PRR3, and WIRS motif for Nck1/Grb2 and the WRC, we performed co-IP and mass spectrometry analysis using DC2.4 infected with *Tg*WIP^WT^, *Tg*WIP^PRR2 mut^, *Tg*WIP^PRR3 mut^, or *Tg*WIP^WIRS mut^ *Toxoplasma*. We included DCs infected with *Tg*WIP^Y150A/Y199A^ parasites as a control. Table 1 lists the identified host binding partners, along with their relative binding strength to *Tg*WIP^WT^, represented as negative fold changes in peptide counts.

**TABLE 1:** Identification of host interaction partners of *Tg*WIP^*a*^

Category	Protein	*Tg*WIP^PRR2 mut^	*Tg*WIP^PRR3 mut^	*Tg*WIP^Y150A/Y199A^	*Tg*WIP^WIRS mut^
SH3-containing proteins	Sorbs1 (Cap)	14	30	1	2
	Sh3rf1	10	6	2	2
	Grb2	6	3	4	2
	Nck1	1	60	1	1
	Nck2	1	3	1	1
Components of the WRC	Abi1	4	6	2	4
	Brk1 (Hspc300)	4	9	1	3
	Cyfip2	3	5	2	2
	Wasf2 (Wave2)	3	15	2	3
	Nckap1l (Hem1)	3	4	1	2
	Abi3	3	7	1	1
	Cyfip1	2	5	1	2
SH2-containing proteins	Ptpn6 (Shp1)	3	3	32	2
	Ptpn11 (Shp2)	3	2	39	2
Novel *Tg*WIP interactors	Pstpip1	8	5	1	1
	Nostrin	6	5	1	3
	Git1	4	11	2	2
	Arhgef7	4	332	3	2
	Git2	2	453	3	2
	Arhgef7	4	8	2	2
	Arhgef6	3	7	2	2
	Lrrk1	3	3	1	1

^*a*^ The DC2.4 cell line was infected with indicated parasites expressing HA-tagged *Tg*WIP at a multiplicity of infection (MOI) of 7. After 4 h of infection, DCs were harvested and lysed. *Tg*WIP was then pulled down using the HA tag. The table represents the host interaction partners identified through mass spectrometry. The values represent negative fold changes in peptide counts for each host protein identified in *Tg*WIP mutant protein immunoprecipitations relative to *Tg*WIP^WT^. Components of the WRC (Cyfip1, Cyfip2, WAVE2, Abi1, Nckap1, and Hspc300/Brick1), the phosphatases Shp1 and Shp2, and the SH3-containing adaptor proteins Nck1/2 and Grb2 were among the identified host proteins.

This analysis revealed previously identified *Tg*WIP interactors, including Shp1, Shp2, Nck1, Nck2, Grb2, and components of the WRC (Cyfip1, Cyfip2, WAVE2, Abi1, Nckap1, and Hspc300/Brick1) (13). Specifically, we observed a stark decrease in Shp1 and Shp2 binding to *Tg*WIP^Y150A/Y199A^, but no change in binding to other *Tg*WIP mutants, consistent with our previous data showing *Tg*WIP’s Y150 and Y199 as primary binding motifs for Shp1 and Shp2 (14). This also confirms that mutations in *Tg*WIP’s PRR do not disturb *Tg*WIP’s ability to become phosphorylated and consequently bind to Shp2 (Fig. 1B and D). Notably, compared to *Tg*WIP^WT^, binding to Nck1 was drastically reduced (60-fold decrease) in *Tg*WIP^PRR3 mut^ but not in *Tg*WIP^PRR2 mut^. Additionally, in contrast to our biochemical analysis, *Tg*WIP^PRR2 mut^ showed a reduction in binding to Grb2, suggesting that PRR2, while not essential for Nck1 interactions, may contribute to Grb2 binding. The interaction with WRC was also reduced in PRR2 and PRR3 mutants, suggesting the *Tg*WIP-WRC interaction *in vivo* may involve additional factors, such as potential interaction of the SH3 domain in Abi1/2/3 with *Tg*WIP’s PRRs or the interaction between WRC and Nck1.

Additionally, new *Tg*WIP interactors were also identified. These include important regulators of the actin cytoskeleton, such as adaptor proteins (SH3P12 and SH3RF1), GTPase-activating proteins (GIT1 and GIT2), and guanine nucleotide exchange factors (ARHGEF7/6) (Table 1) (34–36). These proteins either contain SH3 domains, such as ARHGEF6/7, Sorbin, and SH3RF1, or form a complex with SH3-containing proteins (GIT1/2), suggesting that they could interact with the PRRs of *Tg*WIP. Unlike *Tg*WIP^PRR3^ or *Tg*WIP^Y150A/Y199A^, we did not observe the binding of *Tg*WIP’s interactors to be mediated uniquely by *Tg*WIP^PRR2 mut^. Indeed, *Tg*WIP^PRR2 mut^ only displayed shared interactions with *Tg*WIP^PRR3 mut^, highlighting the dominant role of *Tg*WIP’s PRR3 in mediating *Tg*WIP’s binding partners.

## DISCUSSION

Intracellular pathogens have evolved various mechanisms to hijack the host actin cytoskeleton to promote their invasion, replication, and dissemination. We previously showed that the widespread parasite *Toxoplasma gondii* manipulates the actin cytoskeleton and enhances the motility of infected DCs through the secreted effector *Tg*WIP, which interacts with and activates the host phosphatases Shp1/2 (14). In this study, we identify an additional mechanism by which *Tg*WIP manipulates the host cell actin cytoskeleton. We show that *Tg*WIP directly interacts with the host adaptor proteins Nck1 and Grb2, as well as the actin nucleation-promoting factor WRC, through distinct motifs. Specifically, *Tg*WIP’s PRR1 and PRR3 sequences are crucial for the interaction with Grb2 and Nck1, respectively, while its WIRS motif is critical for interacting with the WRC. Mutating any of these motifs abolishes *Tg*WIP-mediated podosome dissolution, F-actin stress fiber formation, and hypermobility of infected BMDCs.

Nck1 and Grb2 are critical regulators of the actin cytoskeleton. They contain an SH2 domain and multiple SH3 domains, which function as scaffolds to recruit diverse effectors containing PRRs and phosphotyrosines. Among their major effectors are the nucleation-promoting factors WASP and N-WASP, which activate Arp2/3-mediated actin polymerization, essential for filopodia formation, podosome formation, and cell migration. The multivalent interactions between Nck1, WASP/N-WASP, and additional factors, such as Nephrin, are known to drive liquid-liquid phase separation (LLPS), which precisely controls the location and timing of actin assembly (37). Our study raises the intriguing hypothesis that *Tg*WIP mimics the PRRs of WASP/N-WASP, thereby interfering with Nck-WASP/N-WASP LLPS and Arp2/3-dependent actin remodeling through direct competition. Similarly, *Tg*WIP could disrupt the interactions of Nck1 and Grb2 with other host effectors containing PRRs and/or phosphotyrosines, such as Cortactin and Gab1 (Grb2-associated binder 1). Given the promiscuity of SH3-PRR interactions, it remains challenging to precisely define the role of *Tg*WIP in modulating related pathways across different cell types and stages of infection.

Our biochemical analysis revealed differences in *Tg*WIP binding to Nck1 and Grb2 depending on the experiment. IPs from murine DCs identified PRR3 as the dominant motif mediating binding to both Nck1 and Grb2, with PRR1 contributing to a lesser extent. In contrast, GST pull-down assays using recombinant human proteins confirmed PRR3 as the primary domain for Nck1 binding, consistent with the IP data. However, in the case of Grb2, the pull-down assays identified PRR1, not PRR3, as the main mediator of the *Tg*WIP-Grb2 interaction. One possible explanation for this discrepancy is the species differences between the experimental systems: IPs were performed in murine DCs, whereas the *in vitro* assays used human recombinant proteins. Although the orthologs are highly conserved, small differences in SH3 domain sequences could influence PRR binding specificity. Taken together, these results suggest that PRR3 mediates a robust interaction with Nck1, and while it also contributes to Grb2 binding *in vivo*, PRR1 is the dominant mediator of the *Tg*WIP and Grb2 interaction *in vitro*.

The WRC is another key actin nucleation-promoting factor, important for lamellipodia formation, phagocytosis, and cell migration. Acting as a signaling hub between the plasma membrane and the Arp2/3 complex/actin, the WRC interacts directly with various transmembrane proteins, particularly cell-adhesion molecules, containing a conserved WIRS motif. The WIRS motif in *Tg*WIP binds to the WRC robustly, which could compete against WIRS-containing membrane proteins, leading to WRC mislocalization and disruption of WRC-mediated actin assembly. In addition, *Tg*WIP could interact with the WRC through alternative routes, including the potential interaction with the C-terminal SH3 domain of the Abi1/2/3 subunit or through indirect interactions by binding to WRC-associated proteins that contain SH3 domains, such as Nck1. This could explain why, in our mass spectrometry analyses, disruption of *Tg*WIP’s PRRs reduced WRC binding more severely than mutation of the WIRS motif, while in our *in vitro* pull-down assays, the *Tg*WIP-WRC interaction depended exclusively on the WIRS motif. Together, these seemingly conflicting observations suggest *Tg*WIP hijacks WRC-mediated pathways through multiple, potentially redundant mechanisms.

Supporting this, mass spectrometry also identified a broader network of SH3-containing proteins that associate with *Tg*WIP, including Sorbin (also known as CAP), SH3RF1, and several GAPs and GEFs involved in actin regulation. Interestingly, although PRR2 mutations had little effect on Nck1 or Grb2 binding, they phenocopied PRR3 mutations in cytoskeletal assays. This suggests that PRR2 recruits one or more of these additional SH3-domain partners. Sorbin is a particularly compelling candidate, as it is a scaffolding protein known to regulate the formation of F-actin stress fibers and focal adhesions (34, 38). Its interaction with *Tg*WIP via PRR2 may explain how PRR2 mutation results in cytoskeletal phenotypes similar to those regulated by Nck1/Grb2-binding PRR3, reinforcing the idea that multiple SH3-dependent interactions contribute to *Tg*WIP function.

Noteworthy, while our proteomic analysis provides a broader view of host proteins interacting with *Tg*WIP, interpreting the significance of modest fold changes in peptide counts remains a challenge. For instance, although mass spectrometry showed a threefold reduction in Grb2 binding for the PRR3 mutant and a sixfold reduction for the PRR2 mutant, PRR2 retained Grb2 binding both in our co-IP and recombinant protein experiments. This highlights that while mass spectrometry is efficient for identifying potential partners, validating these interactions using pull-down assays remains crucial, especially for interactors depicting low fold changes (under ~10-fold).

While the simplest model to explain the mechanism of *Tg*WIP is that it uses distinct sequence motifs to bind to different host proteins, with each interaction modulating a specific aspect of host cell behavior, our findings suggest a more integrated mechanism. We have defined the motifs mediating *Tg*WIP’s interactions with Shp1/2, Nck1/Grb2, and the WRC. However, it is striking that disrupting any single interaction results in highly similar, pleiotropic effects on the host actin cytoskeleton and cell migration. This suggests that *Tg*WIP functions as part of a highly cooperative process, depending on the synergistic actions of multiple host protein interactions. Disruption of one interaction likely weakens the overall activity of *Tg*WIP, diminishing its ability to fully remodel the host cytoskeleton. Such functional interdependence could explain the strong evolutionary conservation of *Tg*WIP’s important sequence motifs across different *Toxoplasma* strains. Future studies testing double PRR/WIRS mutants could clarify whether their functions are synergistic or redundant.

While our *in vitro* studies have elucidated the molecular mechanisms by which *Tg*WIP remodels the actin cytoskeleton and enhances DC migration, the relevance of these findings to parasite dissemination *in vivo* remains an important question. Previous work showed that *Tg*WIP deletion reduces *Toxoplasma* spread in mice, but it remains unclear whether individual mutations in the PRRs, tyrosine residues, or WIRS motif affect dissemination. Testing these *Tg*WIP mutations in animal models, using approaches, such as *in vivo* imaging or quantifying organ parasite burden, would help understand the impact of specific host-parasite interactions to *Toxoplasma’s* dissemination. This would also clarify the extent to which the hypermigratory phenotype observed *in vitro* contributes to *Toxoplasma’s* dissemination strategy *in vivo*.

*Tg*WIP is one of several *Toxoplasma* effectors that promote leukocyte hypermotility by targeting distinct host actin regulatory pathways. Notably, *Toxoplasma Tg*14-3-3 and ROP17 have also been linked to hypermigration of infected cells. *Tg*14-3-3 is a cytoplasmic and secreted protein with multiple functions in *Apicomplexa*, and its expression in primary DCs and microglia induces a hypermigratory phenotype (19). Although the exact mechanism remains unclear, *Tg*14-3-3 is hypothesized to modulate the Ras-Raf-Mek-Erk MAPK signaling cascade, a pathway implicated in hypermigration of infected DCs (39, 40). This is supported by observations that host 14-3-3 proteins accumulate around the parasitophorous vacuole in infected DCs, suggesting sequestration and modulation of MAPK signaling (19). In contrast, ROP17 is a kinase effector that promotes the migration of infected monocytes by targeting GEFs that regulate Rho-ROCK signaling, a pathway that regulates actin nucleation and contractility (20). The distinct host signaling pathways targeted by *Tg*WIP, *Tg*14-3-3-, and ROP17 underscore the possibility for additive or epistatic interactions among these effectors. Together, they highlight the multilayered strategies *Toxoplasma* utilizes to enhance the migratory capacity of distinct leukocyte cell types and promote parasite dissemination.

**Fig 5:**
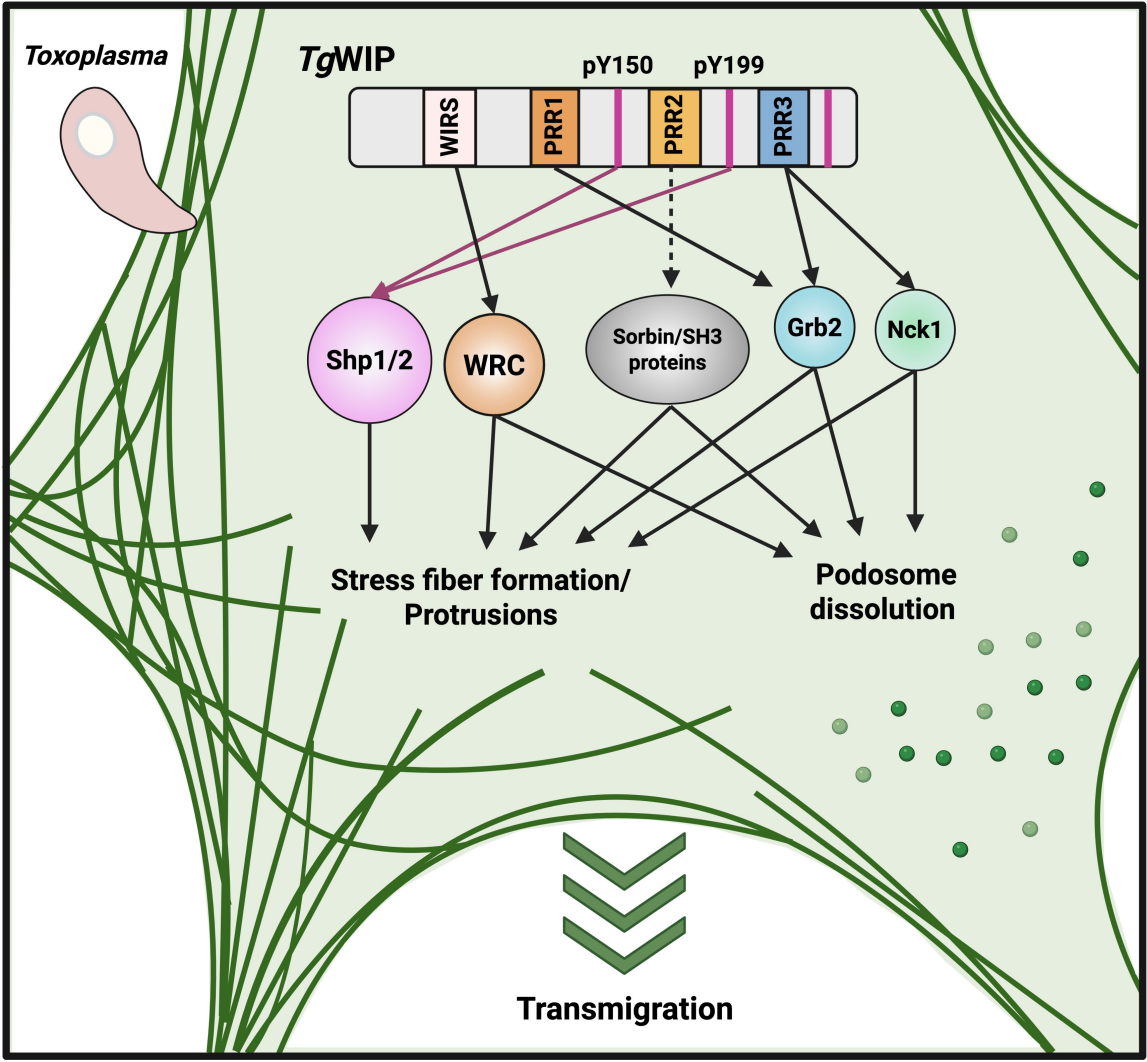
Schematic summary of *Tg*WIP-mediated interactions and their effects on host dendritic cell actin dynamics and migration. *Tg*WIP engages different host proteins via multiple motifs to modulate the actin cytoskeleton and promote DC migration. The WIRS motif binds to components of the WRC, promoting stress fiber formation, membrane protrusions, and podosome dissolution, resulting in enhanced DC transmigration. Phosphorylated tyrosines Y150 and Y199 recruit the tyrosine phosphatases Shp1 and Shp2, further contributing to stress fiber assembly, protrusion formation, and enhanced DC transmigration. The PRR1 binds to Grb2, while PRR3 interacts with both Grb2 and Nck1, each driving stress fiber formation, membrane protrusions, podosome dissolution, and enhanced DC transmigration. PRR2 is hypothesized to bind Sorbin and other SH3 domain-containing proteins, possibly mediating cytoskeletal changes and transmigration enhancement.

In summary (Fig. 5), this study defines a multifaceted mechanism by which the *Toxoplasma gondii* effector *Tg*WIP hijacks the host actin cytoskeleton to promote DC hypermotility. We demonstrate how *Tg*WIP utilizes distinct sequence motifs to interact with multiple host proteins, Shp1/2, Nck1/Grb2, and the WAVE regulatory complex. This mechanism allows *Tg*WIP to employ pleiotropic effects on podosome and F-actin stress fiber formation and cell migration. Our findings place *Tg*WIP alongside other *Toxoplasma* effectors, such as *Tg*14-3-3 and ROP17, that also target host cytoskeletal regulators to promote immune cell hypermotility. These effectors highlight parasite virulence strategies in which *Toxoplasma* hijacks host cytoskeleton networks to disseminate efficiently within the host. This work not only builds on the molecular mechanisms used by intracellular pathogens but also reinforces the known host molecular pathways that govern the actin cytoskeleton and cell motility.

## MATERIALS AND METHODS

### Parasite culture

All the *Toxoplasma* parasite strains were routinely maintained *in vitro* in monolayers of HFF at 37°C in 5% CO_2_ as previously described (41).

### Primary host cell culture

BMDCs were isolated from female CD1 mice (Charles River Laboratories) of 5-to-8 weeks old as previously described (42). BMDCs were obtained by culturing murine bone marrow cells in Roswell Park Memorial Institute 1640 with 5% FBS, 10 mM HEPES (pH 7.9), 50 µM 2-mercaptoethanol, 10 µg/mL gentamicin, referred to as complete medium (CM), and supplemented with recombinant mouse GM-CSF (40 ng/mL, Peprotech) and mouse IL-4 (40 ng/mL, Peprotech). Loosely adherent cells were harvested after 8 days of maturation. The medium was changed every 2 days in culture with fresh GM-CSF and IL-4 (modified from Inaba et al. [43]).

### Co-immunoprecipitation

DC2.4 cells were grown in a 150 mm culture dish until 100% confluency and infected (multiplicity of infection [MOI]: 5 to 7) for 4 h with ME49 *Tg*WIP^WT^ parasite expressing *Tg*WIP mutant strains or mock infected, using an MOI of 5 to 7. Cells were then scraped in PBS, centrifuged, and resuspended in 1 or 3 mL of lysis buffer (HEPES 10 mM, pH 7.9, MgCl_2_ 1.5 mM, KCl 10 mM, EDTA 0.1 mM, dithiothreitol 0.5 mM, NP40 0.65%, cocktail of protease inhibitor [Roche], phenylmethylsulfonyl fluoride [PMSF] 0.5 mM) for 30 min at 4°C. The lysate was centrifuged for 30 min at 18,000 × *g*, 4°C. Each sample was incubated with 35 µL of magnetic beads coupled with HA antibodies (Thermo Scientific) and placed on a rotator overnight at 4°C. The beads were washed three times with Tris-HCl 10 mM, pH 7.5, NaCl 150 mM, Triton X-100 0.2%, PMSF 0.5 mM, a cocktail of protease inhibitors (Roche), followed by one-time wash with Tris-HCl 62.5 mM, pH 6.8, and finally resuspended in 100 µL of this buffer for analyses. Bound proteins were eluted by boiling the samples for 5 min.

### Immunoblotting

For each sample, 30 µL of the HA magnetic beads of each sample was used to run on a 12% SDS-PAGE gel. The proteins were transferred to a PVDF membrane, blocked for 30 min with Tris-buffered saline with Tween 20 containing 5% nonfat dry milk. The membrane was blotted overnight at 4°C with a rat antibody against the HA tag (Sigma Aldrich, Cat#11867431001, add the exact number for all antibodies 1:500 dilution), phosphorylated Tyrosine (Cell Signaling, Cat#8954S, 1:500 dilution), Shp1 (Invitrogen, Cat#3759, 1:1,000 dilution), or Shp2 (Cell Signaling, Cat#3752S, 1:1,000 dilution) antibodies, followed by their respective secondary antibodies (ThermoFisher Scientific, 1:5,000 dilution).

### Proline-rich region mutants generation

The prolines in the PRR regions were replaced by alanines, either individually or in combination, to create single, double, and triple PRR mutants. The sequences were synthesized by Twist Biosciences and incorporated into the complementation plasmid pUPRT::TgWIP-HA (13). *Toxoplasma* ME49 ∆*tgwip* was transfected with the complementation plasmid, using Gene Pulser Xcell (BioRad), as previously described in reference 44. The transfected parasites were selected with 10 µM of FUDR (5-fluorodeoxyuracil) and cloned by limiting dilution.

### Podosome assay

To test the podosome dissolution after *Toxoplasma* challenge, BMDCs were seeded on collagen-coated glass coverslips overnight, after which freshly egressed parasites were added at an MOI of 3 for 4 h. The coverslips were then washed with PBS, fixed with 4% paraformaldehyde (PFA) for 20 min, permeabilized for 5 min with 0.1% Triton X-100, and blocked for 1 h with PBS with 3% (wt/vol) BSA (blocking buffer). The coverslips were incubated with DAPI to stain the host nucleus, and with Alexa Fluor 488 Phalloidin in blocking buffer for 30 min to visualize F-actin. GFP-expressing *Toxoplasma* were detected through their intrinsic GFP signal. The coverslips were mounted with Vecta-Shield mounting oil, and the microscopy was performed with NIS-Elements software (Nikon) and a digital camera (CoolSNAP EZ; Roper Scientific) connected to an inverted fluorescence microscope (Eclipse Ti-S; Nikon) and either phase contrast or DIC imaging. Podosomes were identified and quantified as described in reference 11.

### DC gelatin degradation assay and image analysis

The *in vitro* gelatinolytic activity of DCs was analyzed by gelatinolysis of Oregon green 488 (OG 488)-conjugated porcine gelatin (Molecular Probes). DCs (2.5 × 10^4^/well) infected with freshly egressed *Toxoplasma* tachyzoites (ME49-GFP, MOI 3) were deposited on OG-488 gelatin-coated glass coverslips and incubated for 24 h in CM. Cells were subsequently fixed (4% paraformaldehyde, Sigma), stained with DAPI (Invitrogen) or Alexa Fluor 594 Phalloidin (Thermo Fisher). Imaging and analysis were performed as indicated below. The *in vitro* gelatinolytic activity of DCs was analyzed by gelatinolysis of Cy-3-conjugated gelatin (molecular probes). After fixation (4% PFA, Sigma), cells were stained with DAPI (1:1,000) and Alexa Fluor 594 Phalloidin (1:800). Using the software ImageJ version 1.54, the threshold of the green channel (Alexa Fluor 488 Phalloidin) was adjusted to distinguish single cells. The tool ‘‘Analyze particles’’ was used to count cells and measure the area of degradation. Gelatin degradation was defined as the loss of signal (gelatin, Oregon green-488). The degradation of 100 cells was manually quantified for each condition. The “Analyze particles” tool in ImageJ was used to quantify DC cell area and roundness based on F-actin staining with phalloidin, which outlines the full cell body. The same tool was also used to measure nuclear area using DAPI to visualize nuclei.

### Transmigration assay

The transmigration assays were performed by culturing BMDCs in CM with the addition of freshly egressed *Toxoplasma* strains (MOI 3) for 4 h. DCs were then transferred to transwell filters consisting of a porous membrane (8 µm; Corning) and incubated at 37°C. After 18 h, DCs from the bottom chamber were collected and quantified by hemocytometer.

### Protein purification

See Tables S1 and S2 for the amino acid sequence of all *Tg*WIP constructs used in the pulldown experiments. All GST-3C-*Tg*WIP PRR mutant constructs were synthesized by ThermoFisher GeneArt and sequence verified independently. All *Tg*WIP constructs, as well as Nck1 and Grb2 constructs, were expressed in Arctic Express (DE3) RIL (Agilent) cells, using induction with 1 mM IPTG at 10°C for 16 h. Cells were disrupted by sonication, followed by centrifugation at 19,000 rpm at 4°C for 30 min. Clarified supernatant was mixed with GSH-Sepharose beads (Cytiva) for 30 min at 4°C, followed by three washes and elution using a buffer containing 30 mM reduced GSH and 100 mM Tris, pH 8.5. Eluted proteins were further purified using Source 15S cation chromatography (Cytiva) at pH 8, followed by size exclusion chromatography using a Superdex 200 Increase gel filtration column (Cytiva). The WRC was purified and assembled as previously described (45).

### Pull-down assay

Pull-down assays were performed as previously described (46). Nck1 and Grb2 experiments were performed with 200 pmol of bait (GST-*Tg*WIP), 1,000 pmol of prey (MBP-Nck1 FL or MBP-Grb2), and in 100PDB buffer (100 mM NaCl, 10 mM HEPES, pH 7.0, 5% [wt/vol] glycerol, 5 mM 2-mercaptoethanol, and 0.05% Triton X-100). WRC experiments were performed with 200 pmol of bait (GST-*Tg*WIP) and 60–300 pmol of prey (WRC), and in 100PDB or 50PDB buffer (50 mM NaCl, 10 mM HEPES, pH 7.0, 5% [wt/vol] glycerol, 5 mM 2-mercaptoethanol, and 0.05% Triton X-100). In each reaction, 20 µL of GSH-Sepharose beads (Cytiva) were mixed with bait and prey proteins in 1 mL of pull-down buffer. The samples were mixed at 4°C for 30 min and then washed three times with 1 mL of pull-down buffer. Protein was eluted with 40 µL of elution buffer (containing 30 mM reduced glutathione and 100 mM Tris, pH 8.5), and the eluate was examined by SDS-PAGE and Coomassie blue staining. Pull-down experiments were repeated at least twice to ensure reproducibility of the results.

## Data Availability

All data are shown in the (supplementary) figures and are also available from the corresponding author.

## SUPPLEMENTAL MATERIAL

Supplemental figures and tables mbio.01874-25-s0001.pdf Fig. S1 to S4 and Tables S1 and S2.

ASM does not own the copyrights to Supplemental Material that may be linked to, or accessed through, an article. The authors have granted ASM a non-exclusive, world-wide license to publish the Supplemental Material files. Please contact the corresponding author directly for reuse.
