# Supplementary material for: Toxoplasma effector TgWIP hijacks dendritic cell actin and motility via Nck1/Grb2 and the WAVE complex
Source: mBio. 2025 Oct 31;16(12):e01874-25. doi: 10.1128/mbio.01874-25 (PMC12691643; doi:10.1128/mbio.01874-25)
Supplement: Supplemental figures and tables — Fig. S1 to S4 and Tables S1 and S2. [file mbio.01874-25-s0001.pdf]

Supplemental Figures

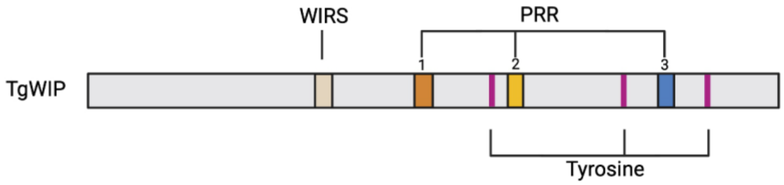

TgWIP WT

MKTETRQRSRGGGKRLSLCVVFALVSISSVSFVTASDQKQGSQNPAGGKGGSGPHGGRRGRQRGVQGGGP
PARPPSPSPEEEPIFGTFVKTDSGGVRGVADSGGNKGRGHHSPHPGPLPPPVPPRLPLRSSSPPSGPRAP
KPQTETSVTYAELQFPQRPPRPPLPPPPGSHGSHSSPTTLGSGAARPHHSVPQPVFSIYATLNTPK
PESPPPVPPPRSVSLLPPSLRSAYPHHPTEDSTGGRGSPSHTRDTGHKKD

TgWIP all mut

MKTETRQRSRGGGKRLSLCVVFALVSISSVSFVTASDQKQGSQNPAGGKGGSGPHGGRRGRQRGVQGGGP
PARAPSPSAEEEPIFGTFVKTDSGGVRGVADSGGNKGRGHHSPHPGPLAAPVAARLALRSSSPPSGARAP
KPQTETSVTYAELQFAQRAPRPALAPPPGSHGSHSSPTTLGSGAARPHHSVAQPVFSIYATLNTPK
AESPAAVPAPRSVSLLAPSLRSAYAHHPTEDSTGGRGSPSHTRDTGHKKD

TgWIP PRR1 mut

MKTETRQRSRGGGKRLSLCVVFALVSISSVSFVTASDQKQGSQNPAGGKGGSGPHGGRRGRQRGVQGGGP
PARPPSPSPEEEPIFGTFVKTDSGGVRGVADSGGNKGRGHHSPHPGPLAAPVAARLALRSSSPPSGARAP
KPQTETSVTYAELQFPQRPPRPPLPPPPGSHGSHSSPTTLGSGAARPHHSVPQPVFSIYATLNTPK
PESPPPVPPPRSVSLLPPSLRSAYPHHPTEDSTGGRGSPSHTRDTGHKKD

TgWIP PRR2 mut

MKTETRQRSRGGGKRLSLCVVFALVSISSVSFVTASDQKQGSQNPAGGKGGSGPHGGRRGRQRGVQGGGP
PARPPSPSPEEEPIFGTFVKTDSGGVRGVADSGGNKGRGHHSPHPGPLPPPVPPRLPLRSSSPPSGPRAP
KPQTETSVTYAELQFAQRAPRPALAPPPGSHGSHSSPTTLGSGAARPHHSVAQPVFSIYATLNTPK
PESPPPVPPPRSVSLLPPSLRSAYPHHPTEDSTGGRGSPSHTRDTGHKKD

TgWIP PRR3 mut

MKTETRQRSRGGGKRLSLCVVFALVSISSVSFVTASDQKQGSQNPAGGKGGSGPHGGRRGRQRGVQGGGP
PARPPSPSPEEEPIFGTFVKTDSGGVRGVADSGGNKGRGHHSPHPGPLPPPVPPRLPLRSSSPPSGPRAP
KPQTETSVTYAELQFPQRPPRPPLPPPPGSHGSHSSPTTLGSGAARPHHSVPQPVFSIYATLNTPK
AESPAAVPAPRSVSLLAPSLRSAYAHHPTEDSTGGRGSPSHTRDTGHKKD

TgWIP PRR1+2 mut

MKTETRQRSRGGGKRLSLCVVFALVSISSVSFVTASDQKQGSQNPAGGKGGSGPHGGRRGRQRGVQGGGP
PARPPSPSPEEEPIFGTFVKTDSGGVRGVADSGGNKGRGHHSPHPGPLAAPVAARLALRSSSPPSGARAP
KPQTETSVTYAELQFAQRAPRPALAPPPGSHGSHSSPTTLGSGAARPHHSVAQPVFSIYATLNTPK
PESPPPVPPPRSVSLLPPSLRSAYPHHPTEDSTGGRGSPSHTRDTGHKKD

TgWIP PRR1+3 mut

MKTETRQRSRGGGKRLSLCVVFALVSISSVSFVTASDQKQGSQNPAGGKGGSGPHGGRRGRQRGVQGGGP
PARPPSPSPEEEPIFGTFVKTDSGGVRGVADSGGNKGRGHHSPHPGPLAAPVAARLALRSSSPPSGARAP
KPQTETSVTYAELQFPQRPPRPPLPPPPGSHGSHSSPTTLGSGAARPHHSVPQPVFSIYATLNTPK
AESPAAVPAPRSVSLLAPSLRSAYAHHPTEDSTGGRGSPSHTRDTGHKKD

TgWIP PRR2+3 mut

MKTETRQRSRGGGKRLSLCVVFALVSISSVSFVTASDQKQGSQNPAGGKGGSGPHGGRRGRQRGVQGGGP
PARPPSPSPEEEPIFGTFVKTDSGGVRGVADSGGNKGRGHHSPHPGPLPPPVPPRLPLRSSSPPSGPRAP
KPQTETSVTYAELQFAQRAPRPALAPPPGSHGSHSSPTTLGSGAARPHHSVAQPVFSIYATLNTPK
AESPAAVPAPRSVSLLAPSLRSAYAHHPTEDSTGGRGSPSHTRDTGHKKD

TgWIP WIRS mut

MKTETRQRSRGGGKRLSLCVVFALVSISSVSFVTASDQKQGSQNPAGGKGGSGPHGGRRGRQRGVQGGGP
PARPPSPSPEEEPIAGAAVKTDSGGVRGVADSGGNKGRGHHSPHPGPLPPPVPPRLPLRSSSPPSGPRAP
KPQTETSVTYAELQFPQRPPRPPLPPPPGSHGSHSSPTTLGSGAARPHHSVPQPVFSIYATLNTPK
PESPPPVPPPRSVSLLPPSLRSAYPHHPTEDSTGGRGSPSHTRDTGHKKD

Fig S1. Alignment of *Tg*WIP amino acid sequences and mutant strains. ME49 *Tg*WIP amino acid sequence of strains *Tg*WIP^{WT}, *Tg*WIP$^{PRR\ all\ mut}$, *Tg*WIP$^{PRR1\ mut}$, *Tg*WIP$^{PRR2\ mut}$, *Tg*WIP$^{PRR3\ mut}$, *Tg*WIP$^{PRR1+2\ mut}$, *Tg*WIP$^{PRR1+3\ mut}$, *Tg*WIP$^{PRR2+3\ mut}$, and *Tg*WIP$^{WIRS\ mut}$. Mutated residues in each strain are indicated. Motifs are color-coded: PRR1 in orange, PRR2 in yellow, PRR3 in blue, tyrosine residues in magenta, WIRS motif in tan.

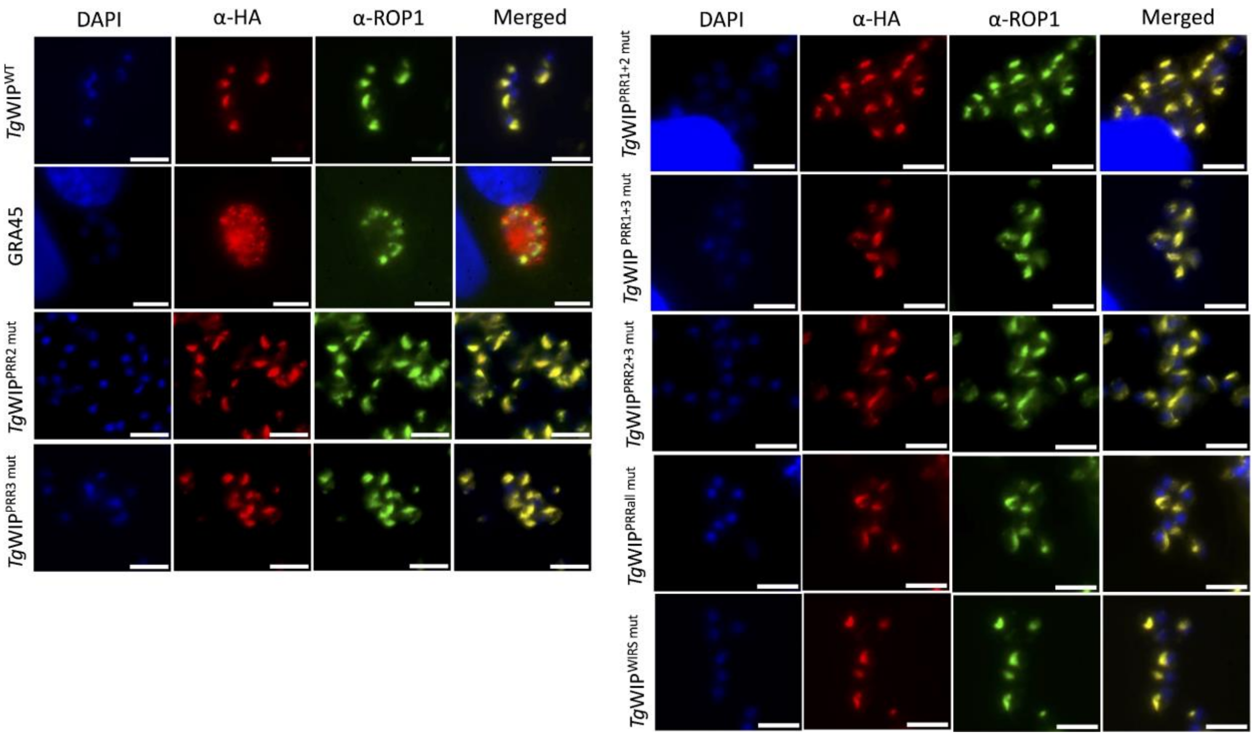

Fig S2. Rhoptry organelle localization of *Tg*WIP mutants. *Tg*WIP and rhoptry organelle colocalization of *Tg*WIP^{WT}, *Tg*WIP^{WIRS}, or *Tg*WIP^{PRR} mutant strains. Immunofluorescence assays on Human Foreskin Fibroblasts (HFFs) infected with *Toxoplasma* containing either endogenously HA-tagged wildtype *Tg*WIP (*Tg*WIP^{WT}) or *Tg*WIP expressing PRR mutations. ROP1 was used as a marker for rhoptry organelles. GRA45 is a *Toxoplasma* secreted protein that localizes in the PV lumen, the GRA45 *Toxoplasma* strain was used as a negative control.

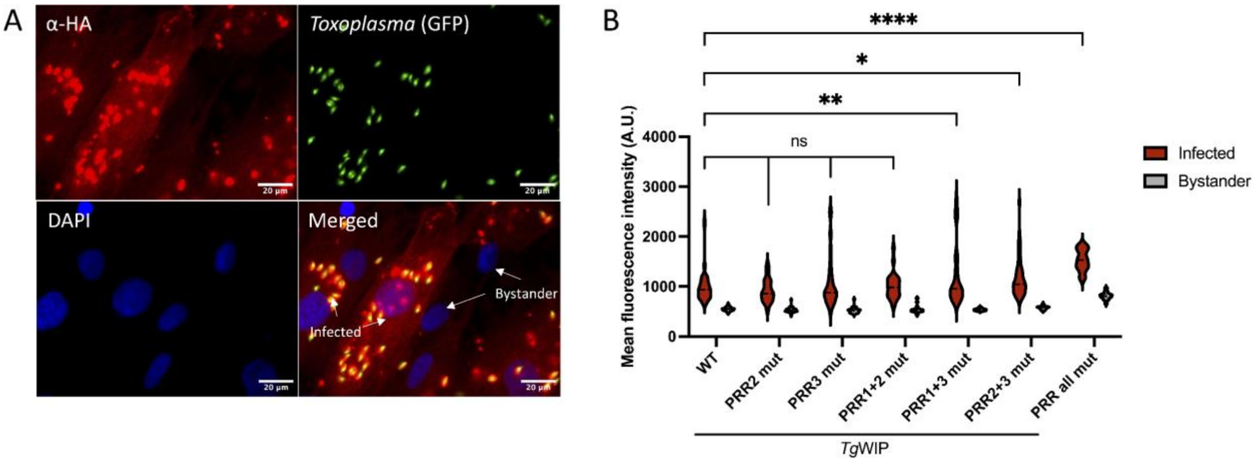

Fig S3. Quantification of *Tg*WIP-HA signal in HFFs infected with *Toxoplasma Tg*WIP^{WT} or *Tg*WIP^{PRR} mutants. A) Representative immunofluorescence images of *Tg*WIP^{WT} *Toxoplasma* infected HFFs stained for HA. White arrows indicate uninfected bystander cells. **B)** The mean HA fluorescence intensity of uninfected (bystander) HFFs or HFFs infected with *Toxoplasma Tg*WIP^{WT} or *Tg*WIP expressing PRR mutations was determined after 3 h infection.

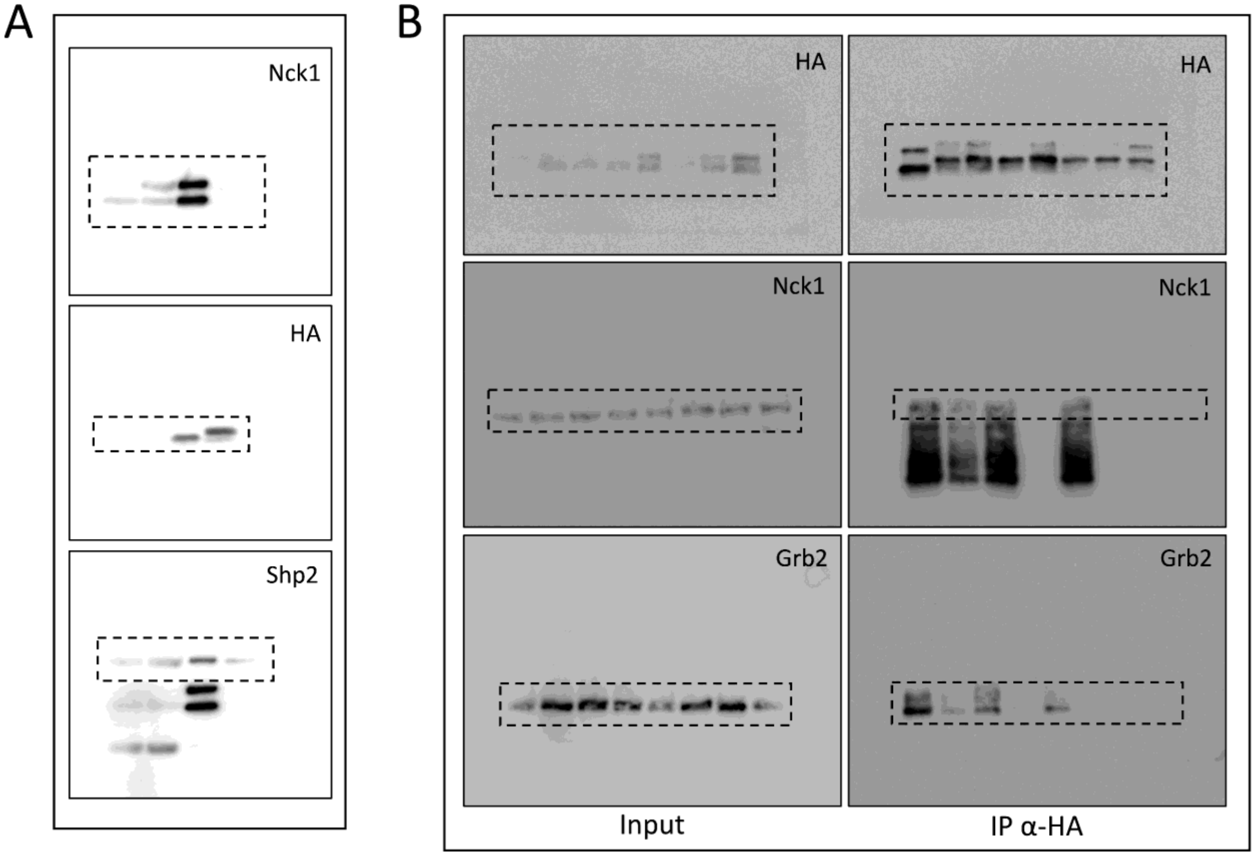

Fig S4. Uncropped Western blot images for main figures. Uncropped Western blot images in this study. **A)** The membrane was sequentially probed with antibodies against Nck1 (first), HA (second, after one stripping step), and Shp2 (third, after a second stripping). **B)** The membrane was first probed with HA antibody, followed by Nck1 (after one stripping step), and Grb2 (after a second stripping).

Name	Description	Source/reference	Identifier
**Recombinant protein preparation**			
GST-3C vector	GST-3C empty vector that TgWIP constructs were cloned into	(PMID: 38977495)	pDK529
GST-3C-TgWIP2 ΔN32 WT	TgWIP2 sequence, missing the NT 32 residue signal peptide, containing a Trp residue for tracking using A280 measurements	This study	pDK502
GST-3C-TgWIP2 ΔN32 PRR1 mut	Mutation of the proline residues of the WT sequence in PRR1 to alanine	This study	pDK503
GST-3C-TgWIP2 ΔN32 PRR2 mut	Mutation of the proline residues of the WT sequence in PRR2 to alanine	This study	pDK504
GST-3C-TgWIP2 ΔN32 PRR3 mut	Mutation of the proline residues of the WT sequence in PRR3 to alanine	This study	pDK505
GST-3C-TgWIP2 ΔN32 PRR1+2 mut	Mutation of the proline residues of the WT sequence in PRR1 and PRR2 to alanine	This study	pDK506
GST-3C-TgWIP2 ΔN32 PRR1+3 mut	Mutation of the proline residues of the WT sequence in PRR1 and PRR3 to alanine	This study	pDK507
GST-3C-TgWIP2 ΔN32 PRR2+3 mut	Mutation of the proline residues of the WT sequence in PRR2 and PRR3 to alanine	This study	pDK508
GST-3C-TgWIP2 ΔN32 PRR all mut	Mutation of the proline residues of the WT sequence in all PRRs to alanine	This study	pDK509
MBP-TEV-hNCK1 FL	MBP-tagged human Nck, full length protein	This study	pDK501
MBP-TEV-hGrb2 FL	MBP-tagged human Grb2, full length protein	This study	pDK517
GST-3C-TgWIP2 ΔN32 WT WIRS mut	TgWIP2 sequence with the WIRS motif TF residues mutated to AA	This study	pDK510
GST-3C-TgWIP ΔN32 WT	TgWIP1 sequence, missing the NT 32 residue signal peptide, containing a Trp residue for tracking using A280 measurements	(PMID: 38977495)	pDK530
GST-3C-TgWIP ΔN32 F1-F11	TgWIP fragments	This study	pAB6-pAB16
MBP-hHSPC300	MBP-tagged human HSPC300 sequence, for assembly into the WRC	(PMID: 24439376)	pDK069
MBP-hWAVE1 1-178	MBP-tagged human WAVE1 sequence, from residues 1 to 178, for assembly into the WRC	(PMID: 24439376)	pDK120
MBP-hAbi2 1-158	MBP-tagged human Abi2 sequence, from residues 1 to 158, for assembly into the WRC	(PMID: 24439376)	pDK075
His6-hSra1	His6-tagged human Sra1, for assembly into the WRC	(PMID: 24439376)	pDK116
hNap1	Human Nap1 sequence, for assembly into the WRC	(PMID: 24439376)	pDK149

Supplementary Table 1. DNA constructs used in this study.

GST

MSPILGYWKIKGLVQPTRLLLEYLEEKYEEHLYERDEGDKWRNKKFELGLEFPNLPYYIDGDVKLTQSMAIIRYIADKHNMLGGCPK<br>ERAEISMLEGAVLDIRYGVSRIAYSKDFETLKVDFLSKLPEMLKMFEDRLCHKTYLNGDHVTHPDFMLYDALDVVLYMDPMCLDA<br>FPKLVCFKKRIEAIPQIDKYLKSSKYIAWPLQGWQATFGGGDHPPKSDLVPRGSLEVLFQGPHM
GST-TgWIP2 WT<br>MSPILGYWKIKGLVQPTRLLLEYLEEKYEEHLYERDEGDKWRNKKFELGLEFPNLPYYIDGDVKLTQSMAIIRYIADKHNMLGGCPK<br>ERAEISMLEGAVLDIRYGVSRIAYSKDFETLKVDFLSKLPEMLKMFEDRLCHKTYLNGDHVTHPDFMLYDALDVVLYMDPMCLDA<br>FPKLVCFKKRIEAIPQIDKYLKSSKYIAWPLQGWQATFGGGDHPPKSDLVPRGSLEVLFQGPHMWTASDQKQGSQNPAGGKGGSGP<br>HGGRRGRQRGVQGGGPPARPPSPSPEEEPIFGTFVKTDSGGVRGVADSGGNKGRGHHSPHPGPLPPPVPPRLPLRSSSPPSGPRAPKPQ<br>TETSVTYAELQFPQRPPRPPLPPPPGSHGSHSSPTTLGSGAARPHHSVPQPVFSIYATLNTPKPESPPPVPPPRSVSLLPPSLRSAYPHHPT<br>EDSTGGRGSPSHTRDTGHKKD
GST-TgWIP2 PRR1 mut<br>MSPILGYWKIKGLVQPTRLLLEYLEEKYEEHLYERDEGDKWRNKKFELGLEFPNLPYYIDGDVKLTQSMAIIRYIADKHNMLGGCPK<br>ERAEISMLEGAVLDIRYGVSRIAYSKDFETLKVDFLSKLPEMLKMFEDRLCHKTYLNGDHVTHPDFMLYDALDVVLYMDPMCLDA<br>FPKLVCFKKRIEAIPQIDKYLKSSKYIAWPLQGWQATFGGGDHPPKSDLVPRGSLEVLFQGPHMWTASDQKQGSQNPAGGKGGSGP<br>HGGRRGRQRGVQGGGPPARPPSPSPEEEPIFGTFVKTDSGGVRGVADSGGNKGRGHHSPHPGPL**AA**PV**AA**RL**A**LRSSSPPSG**A**RAPK<br>PQTETSVTYAELQFPQRPPRPPLPPPPGSHGSHSSPTTLGSGAARPHHSVPQPVFSIYATLNTPKPESPPPVPPPRSVSLLPPSLRSAYPHH<br>PTEDSTGGRGSPSHTRDTGHKKD
GST-TgWIP2 PRR2 mut<br>MSPILGYWKIKGLVQPTRLLLEYLEEKYEEHLYERDEGDKWRNKKFELGLEFPNLPYYIDGDVKLTQSMAIIRYIADKHNMLGGCPK<br>ERAEISMLEGAVLDIRYGVSRIAYSKDFETLKVDFLSKLPEMLKMFEDRLCHKTYLNGDHVTHPDFMLYDALDVVLYMDPMCLDA<br>FPKLVCFKKRIEAIPQIDKYLKSSKYIAWPLQGWQATFGGGDHPPKSDLVPRGSLEVLFQGPHMWTASDQKQGSQNPAGGKGGSGP<br>HGGRRGRQRGVQGGGPPARPPSPSPEEEPIFGTFVKTDSGGVRGVADSGGNKGRGHHSPHPGPLPPPVPPRLPLRSSSPPSGPRAPKPQ<br>TETSVTYAELQF**A**QR**A**PRP**A**L**A**PPPGSHGSHSSPTTLGSGAARPHHSV**A**QPVFSIYATLNTPKPESPPPVPPPRSVSLLPPSLRSAYPHH<br>PTEDSTGGRGSPSHTRDTGHKKD
GST-TgWIP2 PRR3 mut<br>MSPILGYWKIKGLVQPTRLLLEYLEEKYEEHLYERDEGDKWRNKKFELGLEFPNLPYYIDGDVKLTQSMAIIRYIADKHNMLGGCPK<br>ERAEISMLEGAVLDIRYGVSRIAYSKDFETLKVDFLSKLPEMLKMFEDRLCHKTYLNGDHVTHPDFMLYDALDVVLYMDPMCLDA<br>FPKLVCFKKRIEAIPQIDKYLKSSKYIAWPLQGWQATFGGGDHPPKSDLVPRGSLEVLFQGPHMWTASDQKQGSQNPAGGKGGSGP<br>HGGRRGRQRGVQGGGPPARPPSPSPEEEPIFGTFVKTDSGGVRGVADSGGNKGRGHHSPHPGPLPPPVPPRLPLRSSSPPSGPRAPKPQ<br>TETSVTYAELQFPQRPPRPPLPPPPGSHGSHSSPTTLGSGAARPHHSVPQPVFSIYATLNTPK**A**ESP**AA**VP**A**PRSVSLL**A**PSLRSAY**A**HH<br>PTEDSTGGRGSPSHTRDTGHKKD
GST-TgWIP2 PRR1+2 mut

MSPILGYWKIKGLVQPTRLLLEYLEEKYEEHLYERDEGDKWRNKKFELGLEFPNLPYYIDGDVKLTQSMAIIRYIADKHNMLGGCPK<br>ERAEISMLEGAVLDIRYGVSRIAYSKDFETLKVDFLSKLPEMLKMFEDRLCHKTYLNGDHVTHPDFMLYDALDVVLYMDPMCLDA<br>FPKLVCFKKRIEAIPQIDKYLKSSKYIAWPLQGWQATFGGGDHPPKSDLVPRGSLEVLFQGPHMWTASDQKQGSQNPAGGKGGSGP<br>HGGRRGRQRGVQGGGPPARPPSPSPEEEPIFGTFVKTDSGGVRGVADSGGNKGRGHHSPHPGPL**AA**PV**AA**RL**A**LRSSSPPSG**A**RAPK<br>PQTETSVTYAELQF**A**QR**A**PRP**A**L**A**PPPGSHGSHSSPTTLGSGAARPHHSV**A**QPVFSIYATLNTPKPESPPPVPPPRSVSLLPPSLRSAYP<br>HHPTEDSTGGRGSPSHTRDTGHKKD
GST-TgWIP2 PRR1+3 mut<br>MSPILGYWKIKGLVQPTRLLLEYLEEKYEEHLYERDEGDKWRNKKFELGLEFPNLPYYIDGDVKLTQSMAIIRYIADKHNMLGGCPK<br>ERAEISMLEGAVLDIRYGVSRIAYSKDFETLKVDFLSKLPEMLKMFEDRLCHKTYLNGDHVTHPDFMLYDALDVVLYMDPMCLDA<br>FPKLVCFKKRIEAIPQIDKYLKSSKYIAWPLQGWQATFGGGDHPPKSDLVPRGSLEVLFQGPHMWTASDQKQGSQNPAGGKGGSGP<br>HGGRRGRQRGVQGGGPPARPPSPSPEEEPIFGTFVKTDSGGVRGVADSGGNKGRGHHSPHPGPL**AA**PV**AA**RL**A**LRSSSPPSG**A**RAPK<br>PQTETSVTYAELQFPQRPPRPPLPPPPGSHGSHSSPTTLGSGAARPHHSVPQPVFSIYATLNTPK**A**ESP**AA**VP**A**PRSVSLL**A**PSLRSAY**A**<br>HHPTEDSTGGRGSPSHTRDTGHKKD
GST-TgWIP2 PRR2+3 mut<br>MSPILGYWKIKGLVQPTRLLLEYLEEKYEEHLYERDEGDKWRNKKFELGLEFPNLPYYIDGDVKLTQSMAIIRYIADKHNMLGGCPK<br>ERAEISMLEGAVLDIRYGVSRIAYSKDFETLKVDFLSKLPEMLKMFEDRLCHKTYLNGDHVTHPDFMLYDALDVVLYMDPMCLDA<br>FPKLVCFKKRIEAIPQIDKYLKSSKYIAWPLQGWQATFGGGDHPPKSDLVPRGSLEVLFQGPHMWTASDQKQGSQNPAGGKGGSGP<br>HGGRRGRQRGVQGGGPPARPPSPSPEEEPIFGTFVKTDSGGVRGVADSGGNKGRGHHSPHPGPLPPPVPPRLPLRSSSPPSGPRAPKPQ<br>TETSVTYAELQF**A**QR**A**PRP**A**L**A**PPPGSHGSHSSPTTLGSGAARPHHSV**A**QPVFSIYATLNTPK**A**ESP**AA**VP**A**PRSVSLL**A**PSLRSAY**A**H<br>HPTEDSTGGRGSPSHTRDTGHKKD
GST-TgWIP2 PRR all mut<br>MSPILGYWKIKGLVQPTRLLLEYLEEKYEEHLYERDEGDKWRNKKFELGLEFPNLPYYIDGDVKLTQSMAIIRYIADKHNMLGGCPK<br>ERAEISMLEGAVLDIRYGVSRIAYSKDFETLKVDFLSKLPEMLKMFEDRLCHKTYLNGDHVTHPDFMLYDALDVVLYMDPMCLDA<br>FPKLVCFKKRIEAIPQIDKYLKSSKYIAWPLQGWQATFGGGDHPPKSDLVPRGSLEVLFQGPHMWTASDQKQGSQNPAGGKGGSGP<br>HGGRRGRQRGVQGGGPPARPPSPSPEEEPIFGTFVKTDSGGVRGVADSGGNKGRGHHSPHPGPLPPPVPPRLPLRSSSPPSGPRAPKPQ<br>TETSVTYAELQF**A**QR**A**PRP**A**L**A**PPPGSHGSHSSPTTLGSGAARPHHSV**A**QPVFSIYATLNTPK**A**ESP**AA**VP**A**PRSVSLL**A**PSLRSAY**A**H<br>HPTEDSTGGRGSPSHTRDTGHKKD
MBP-NCK1 FL<br>MKIEEGKLVIWINGDKGYNGLAEVGKKFEKDTGIKVTVEHPDKLEEKFPQVAATGDGPDIIFWAHDRFGGYAQSGLLAEITPDKAFQ<br>DKLYPFTWDAVRYNGKLIAYPIAVEALSLIYNKDLLPNPPKTWEEIPALDKELKAKGKSALMFNLQEPYFTWPLIAADGGYAFKYEN<br>GKYDIKDVGVDNAGAKAGLTFLVDLIKNKHMNADTDYSIAEAAFNKGETAMTINGPWAWSNIDTSKVNYGVTVLPTFKGQPSKPF<br>VGVLSAGINAASPNKELAKEFLENYLLTDEGLEAVNKDKPLGAVALKSYEEELAKDPRIAATMENAQKGEIMPNIPQMSAFWYAVR<br>TAVINAASGRQTVDEALKDAQTNSSSNNNNNNNNNNLGIEGRISEFENLYFQGMAEEVVVVAKFDYVAQQEQELDIKKNERLWLL<br>DDSKSWWRVRNSMNKTGFVPSNYVERKNSARKASIVKNLKDTLGIGKVKRKPSVPDSASPADDSFVDPGERLYDLNMPAYVKFNY

MAEREDELSLIKGTKVIVMEKCSDGWWRGSYNGQVGWFPSNYVTEEGDSPLGDHVGSLSEKLAAVVNNLNTGQVLHVVQALYPF<br>SSSNDEELNFEKGDVMDVIEKPENDPEWWKCRKINGMVGLVPKNYVTVMQNNPLTSGLEPSPPQCDYIRPSLTGKFAGNPWYYGK<br>VTRHQAEMALNERGHEGDFLIRDSESSPNDFSVSLKAQGKNKHFKVQLKETVYCIGQRKFSTMEELVEHYKKAPIFTSEQGEKLYLV<br>KHLS
MBP-Grb2<br>MKIEEGKLVIWINGDKGYNGLAEVGKKFEKDTGIKVTVEHPDKLEEKFPQVAATGDGPDIIFWAHDRFGGYAQSGLLAEITPDKAFQ<br>DKLYPFTWDAVRYNGKLIAYPIAVEALSLIYNKDLLPNPPKTWEEIPALDKELKAKGKSALMFNLQEPYFTWPLIAADGGYAFKYEN<br>GKYDIKDVGVDNAGAKAGLTFLVDLIKNKHMNADTDYSIAEAAFNKGETAMTINGPWAWSNIDTSKVNYGVTVLPTFKGQPSKPF<br>VGVLSAGINAASPNKELAKEFLENYLLTDEGLEAVNKDKPLGAVALKSYEEELAKDPRIAATMENAQKGEIMPNIPQMSAFWYAVR<br>TAVINAASGRQTVDEALKDAQTNSSSNNNNNNNNNNLGIEGRISEFENLYFQGMEAIAKYDFKATADDELSFKRGDILKVLNEECDQ<br>NWYKAELNGKDGFIPKNYIEMKPHPWFFGKIPRAKAEEMLSKQRHDGAFLIRESESAPGDFSLSVKFGNDVQHFKVLRDGAGKYFL<br>WVVKFNSLNELVDYHRSTSVSRNQQIFLRDIEQVPQQPTYVQALFDFDPQEDGELGFRRGDFIHVMDNSDPNWWKGACHGQTGMF<br>PRNYVTPVNRNV
GST-TgWIP2 WIRS mut<br>MSPILGYWKIKGLVQPTRLLLEYLEEKYEEHLYERDEGDKWRNKKFELGLEFPNLPYYIDGDVKLTQSMAIIRYIADKHNMLGGCPK<br>ERAEISMLEGAVLDIRYGVSRIAYSKDFETLKVDFLSKLPEMLKMFEDRLCHKTYLNGDHVTHPDFMLYDALDVVLYMDPMCLDA<br>FPKLVCFKKRIEAIPQIDKYLKSSKYIAWPLQGWQATFGGGDHPPKSDLVPRGSLEVLFQGPHMWTASDQKQGSQNPAGGKGGSGP<br>HGGRRGRQRGVQGGGPPARPPSPSPEEEPIFG**AA**VKTDSGGVRGVADSGGNKGRGHHSPHPGPLPPPVPPRLPLRSSSPPSGPRAPKP<br>QTETSVTYAELQFPQRPPRPPLPPPPGSHGSHSSPTTLGSGAARPHHSVPQPVFSIYATLNTPKPESPPPVPPPRSVSLLPPSLRSAYPHHP<br>TEDSTGGRGSPSHTRDTGHKKD
GST-TgWIP WT<br>MSPILGYWKIKGLVQPTRLLLEYLEEKYEEHLYERDEGDKWRNKKFELGLEFPNLPYYIDGDVKLTQSMAIIRYIADKHNMLGGCPK<br>ERAEISMLEGAVLDIRYGVSRIAYSKDFETLKVDFLSKLPEMLKMFEDRLCHKTYLNGDHVTHPDFMLYDALDVVLYMDPMCLDA<br>FPKLVCFKKRIEAIPQIDKYLKSSKYIAWPLQGWQATFGGGDHPPKSDLVPRGSLEVLFQGPHMWTASDQKQGSQNPAGGKGGSGP<br>HGGRRGRQRGVQGGGPPARPPSPSPEEEPIFGTFVKTDGGGVRGVADSGGNKGRGHHSPHPGPLPPPVPPRLPLRSSSPPSGPRAPKP<br>QTDPTVTYAELQFPQRPPRPPLPPSPGSHGSHSSPTTPGSGAPRPHHSVPQSVSSIYATLNTPKPESPPPVPPPRSVSLLPPSLRSAYPHHP<br>TEDSTGGGGSPSHTRDTGHKKD
GST-TgWIP F1 (original F2)<br>MSPILGYWKIKGLVQPTRLLLEYLEEKYEEHLYERDEGDKWRNKKFELGLEFPNLPYYIDGDVKLTQSMAIIRYIADKHNMLGGCPK<br>ERAEISMLEGAVLDIRYGVSRIAYSKDFETLKVDFLSKLPEMLKMFEDRLCHKTYLNGDHVTHPDFMLYDALDVVLYMDPMCLDA<br>FPKLVCFKKRIEAIPQIDKYLKSSKYIAWPLQGWQATFGGGDHPPKSDLVPRGSLEVLFQGPHMHGGRRGRQRGVQGGGPPARPPSP<br>SPEEEPIFGTFVKTDGGGVRGVADSGGNKGRGHHS
GST-TgWIP F2 (original F3)

MSPILGYWKIKGLVQPTRLLLEYLEEKYEEHLYERDEGDKWRNKKFELGLEFPNLPYYIDGDVKLTQSMAIIRYIADKHNMLGGCPK<br>ERAEISMLEGAVLDIRYGVSRIAYSKDFETLKVDFLSKLPEMLKMFEDRLCHKTYLNGDHVTHPDFMLYDALDVVLYMDPMCLDA<br>FPKLVCFKKRIEAIPQIDKYLKSSKYIAWPLQGWQATFGGGDHPPKSDLVPRGSLEVLFQGPHMTDGGGVRGVADSGGNKGRGHHS<br>PHPGPLPPPVPPRLPLRSSSPPSGPRAPKPQTDPTVTYAELQF
GST-TgWIP F3 (original F4)<br>MSPILGYWKIKGLVQPTRLLLEYLEEKYEEHLYERDEGDKWRNKKFELGLEFPNLPYYIDGDVKLTQSMAIIRYIADKHNMLGGCPK<br>ERAEISMLEGAVLDIRYGVSRIAYSKDFETLKVDFLSKLPEMLKMFEDRLCHKTYLNGDHVTHPDFMLYDALDVVLYMDPMCLDA<br>FPKLVCFKKRIEAIPQIDKYLKSSKYIAWPLQGWQATFGGGDHPPKSDLVPRGSLEVLFQGPHMTDGGGVRGVADSGGNKGRGHHS<br>PHPGPLPPPVPPRLPLRSSS
GST-TgWIP F4 (original F5)<br>MSPILGYWKIKGLVQPTRLLLEYLEEKYEEHLYERDEGDKWRNKKFELGLEFPNLPYYIDGDVKLTQSMAIIRYIADKHNMLGGCPK<br>ERAEISMLEGAVLDIRYGVSRIAYSKDFETLKVDFLSKLPEMLKMFEDRLCHKTYLNGDHVTHPDFMLYDALDVVLYMDPMCLDA<br>FPKLVCFKKRIEAIPQIDKYLKSSKYIAWPLQGWQATFGGGDHPPKSDLVPRGSLEVLFQGPHMRLPLRSSSPPSGPRAPKPQTDPTVT<br>YAELQF
GST-TgWIP F5 (original F6)<br>MSPILGYWKIKGLVQPTRLLLEYLEEKYEEHLYERDEGDKWRNKKFELGLEFPNLPYYIDGDVKLTQSMAIIRYIADKHNMLGGCPK<br>ERAEISMLEGAVLDIRYGVSRIAYSKDFETLKVDFLSKLPEMLKMFEDRLCHKTYLNGDHVTHPDFMLYDALDVVLYMDPMCLDA<br>FPKLVCFKKRIEAIPQIDKYLKSSKYIAWPLQGWQATFGGGDHPPKSDLVPRGSLEVLFQGPHMLRSSSPPSGPRAPKPQTDPTVTYA<br>ELQFPQRPPRPPLPPSPGSHGSHSS
GST-TgWIP F6 (original F7)<br>MSPILGYWKIKGLVQPTRLLLEYLEEKYEEHLYERDEGDKWRNKKFELGLEFPNLPYYIDGDVKLTQSMAIIRYIADKHNMLGGCPK<br>ERAEISMLEGAVLDIRYGVSRIAYSKDFETLKVDFLSKLPEMLKMFEDRLCHKTYLNGDHVTHPDFMLYDALDVVLYMDPMCLDA<br>FPKLVCFKKRIEAIPQIDKYLKSSKYIAWPLQGWQATFGGGDHPPKSDLVPRGSLEVLFQGPHMTVTYAELQFPQRPPRPPLPPSPGSH<br>GSHSS
GST-TgWIP F7 (original F8)<br>MSPILGYWKIKGLVQPTRLLLEYLEEKYEEHLYERDEGDKWRNKKFELGLEFPNLPYYIDGDVKLTQSMAIIRYIADKHNMLGGCPK<br>ERAEISMLEGAVLDIRYGVSRIAYSKDFETLKVDFLSKLPEMLKMFEDRLCHKTYLNGDHVTHPDFMLYDALDVVLYMDPMCLDA<br>FPKLVCFKKRIEAIPQIDKYLKSSKYIAWPLQGWQATFGGGDHPPKSDLVPRGSLEVLFQGPHMTVTYAELQFPQRPPRPPLPPSPGSH<br>GSHSSPTTPGSGAPRPHHSVPQSVSSIYATLNT
GST-TgWIP F8 (original F9)<br>MSPILGYWKIKGLVQPTRLLLEYLEEKYEEHLYERDEGDKWRNKKFELGLEFPNLPYYIDGDVKLTQSMAIIRYIADKHNMLGGCPK<br>ERAEISMLEGAVLDIRYGVSRIAYSKDFETLKVDFLSKLPEMLKMFEDRLCHKTYLNGDHVTHPDFMLYDALDVVLYMDPMCLDA<br>FPKLVCFKKRIEAIPQIDKYLKSSKYIAWPLQGWQATFGGGDHPPKSDLVPRGSLEVLFQGPHMGSHGSHSSPTTPGSGAPRPHHSVP<br>QSVSSIYATLNTPKPESPPPVPPPRSVSLL

GST-TgWIP F9 (original F10)<br>MSPILGYWKIKGLVQPTRLLLEYLEEKYEEHLYERDEGDKWRNKKFELGLEFPNLPYYIDGDVKLTQSMAIIRYIADKHNMLGGCPKERAEISMLEGAVLDIRYGVSRIAYSKDFETLKVDFLSKLPEMLKMFEDRLCHKTYLNGDHVTHPDFMLYDALDVVLYMDPMCLDAFPKLVCFKKRIEAIPQIDKYLKSSKYIAWPLQGWQATFGGGDHPPKSDLVPRGSLEVLFQGPHMQSVSSIYATLNTPKPESPPPVPPPRSVSLLPPSLRSAYPHHPTEDSTGGGGSPSHTRDTGHKKD
GST-TgWIP F10 (original F11)<br>MSPILGYWKIKGLVQPTRLLLEYLEEKYEEHLYERDEGDKWRNKKFELGLEFPNLPYYIDGDVKLTQSMAIIRYIADKHNMLGGCPKERAEISMLEGAVLDIRYGVSRIAYSKDFETLKVDFLSKLPEMLKMFEDRLCHKTYLNGDHVTHPDFMLYDALDVVLYMDPMCLDAFPKLVCFKKRIEAIPQIDKYLKSSKYIAWPLQGWQATFGGGDHPPKSDLVPRGSLEVLFQGPHMQSVSSIYATLNTPKPESPPPVPPPRSVSLL
GST-TgWIP F11 (original F12)<br>MSPILGYWKIKGLVQPTRLLLEYLEEKYEEHLYERDEGDKWRNKKFELGLEFPNLPYYIDGDVKLTQSMAIIRYIADKHNMLGGCPKERAEISMLEGAVLDIRYGVSRIAYSKDFETLKVDFLSKLPEMLKMFEDRLCHKTYLNGDHVTHPDFMLYDALDVVLYMDPMCLDAFPKLVCFKKRIEAIPQIDKYLKSSKYIAWPLQGWQATFGGGDHPPKSDLVPRGSLEVLFQGPHMRSVSLLPPSLRSAYPHHPTEDSTGGGGSPSHTRDTGHKKD
HSPC300<br>GHMGAAMAGQEDPVQREIHQDWANREYIEIITSSIKKIADFLNSFDMSCRSRLATLNEKLTALERRIEYIEARVTKGETLT
WAVE1 (1-178)<br>GHMPLVKRNIDPRHLCHTALPRGIKNELECVTNISLANIIRQLSSLSKYAEDIFGELFNEAHSFSFRVNSLQERVDRLSVSVTQLDPKEEELSLQDITMRKAFRSSTIQDQQLFDRKTLPIPLQETYDVCEQPPPLNILTPYRDDGKEGLKFYTNPSYFFDLWKEKMLQDTEDKRKEKRK
Abi2 (1-158)<br>GHMAELQMLLEEEIPGGRRALFDSYTNLERVADYCENNYIQSADKQRALEETKAYTTQSLASVAYLINTLANNVLQMLDIQASQLRRMESSINHISQTVDIHKEKVARREIGILTTNKNTSRTHKIIAPANLERPVRYIRKPIDYTILDDIGHGVKVSTQ
Sra1<br>GAMAAQVTLEDALSNVDLLEELPLPDQQPCIEPPPSSLLYQPNFNTNFEDRNAFVTGIARYIEQATVHSSMNEMLEEGQEYAVMLYTWRSCSRAIPQVKCNEQPNRVEIYEKTVEVLEPEVTKLMNFMYFQRNAIERFCGEVRRLCHAERRKDFVSEAYLITLGKFINMFAVLDELKNMKCSVKNDHSAYKRAAQFLRKMADPQSIQESQNLSMFLANHNKITQSLQQQLEVISGYEELLADIVNLCVDYYENRMYLTPSEKHMLLKVMGFGLYLMDGSVSNIYKLDAKKRINLSKIDKYFKQLQVVPLFGDMQIELARYIKTSAHYEENKSRWTCTSSGSSPQYNICEQMIQIREDHMRFISELARYSNSEVVTGSGRQEAQKTDAEYRKLFDLALQGLQLLSQWSAHVMEVYSWKLVHPTDKYSNKDCPDSAEEYERATRYNYTSEEKFALVEVIAMIKGLQVLMGRMESVFNHAIRHTVYAALQDFSQVTLREPLRQAIKKKKNVIQSVLQAIRKTVCDWETGHEPFNDPALRGEKDPKSGFDIKVPRRAVGPSSTQLYMVRTMLESLIADKSGSKKTLRSSLEGPTILDIEKFHRESFFYTHLINFSETLQQCCDLSQLWFREFFLELTMGRRIQFPIEMSMPWILTDHILETKEASMMEYVLYSLDLYNDSAHYALTRFNKQFLYDEIEAEVNLCFDQFVYKLADQIFAYYKVMAGSLLLDKRLRSECKNQGATIHLPPSNRYETLLKQRHVQLLGRSIDLNRLITQRVSAAMYKSLE

LAIGRFESEDLTSIVELDGLLEINRMTHKLLSRYLTLDGFDAMFREANHNVSAPYGRITLHVFWELNYDFLPNYCYNGSTNRFVRTVL<br>PFSQEFQRDKQPNAQPQYLHGSKALNLAYSSIYGSYRNFVGPPHFQVICRLLGYQGIAVVMEELLKVVKSLLQGTILQYVKTLMEVM<br>PKICRLPRHEYGSPGILEFFHHQLKDIVEYAELKTVCFQNLREVGNAILFCLLIEQSLSLEEVCDLLHAAPFQNILPRVHVKEGERLDAK<br>MKRLESKYAPLHLVPLIERLGTPQQIAIAREGDLLTKERLCCGLSMFEVILTRIRSFLDDPIWRGPLPSNGVMHVDECVEFHRLWSAM<br>QFVYCIPVGTHEFTVEQCFGDGLHWAGCMIIVLLGQQRRFAVLDFCYHLLKVQKHDGKDEIIKNVPLKKMVERIRKFQILNDEIITIL<br>DKYLKSGDGEGTPVEHVRCFQPPIHQSLASS
Nap1<br>MSRSVLQPSQQKLAEKLTILNDRGVGMLTRLYNIKKACGDPKAKPSYLIDKNLESAVKFIVRKFPAVETRNNNQQLAQLQKEKSEIL<br>KNLALYYFTFVDVMEFKDHVCELLNTIDVCQVFFDITVNFDLTKNYLDLIITYTTLMILLSRIEERKAIIGLYNYAHEMTHGASDREYP<br>RLGQMIVDYENPLKKMMEEFVPHSKSLSDALISLQMVYPRRNLSADQWRNAQLLSLISAPSTMLNPAQSDTMPCEYLSLDAMEKWI<br>IFGFILCHGILNTDATALNLWKLALQSSSCLSLFRDEVFHIHKAAEDLFVNIRGYNKRINDIRECKEAAVSHAGSMHRERRKFLRSALK<br>ELATVLSDQPGLLGPKALFVFMALSFARDEIIWLLRHADNMPKKSADDFIDKHIAELIFYMEELRAHVRKYGPVMQRYYVQYLSGF<br>DAVVLNELVQNLSVCPEDESIIMSSFVNTMTSLSVKQVEDGEVFDFRGMRLDWFRLQAYTSVSKASLGLADHRELGKMMNTIIFHT<br>KMVDSLVEMLVETSDLSIFCFYSRAFEKMFQQCLELPSQSRYSIAFPLLCTHFMSCTHELCPEERHHIGDRSLSLCNMFLDEMAKQAR<br>NLITDICTEQCTLSDQLLPKHCAKTISQAVNKKSKKQTGKKGEPEREKPGVESMRKNRLVVTNLDKLHTALSELCFSINYVPNMVVW<br>EHTFTPREYLTSHLEIRFTKSIVGMTMYNQATQEIAKPSELLTSVRAYMTVLQSIENYVQIDITRVFNNVLLQQTQHLDSHGEPTITSL<br>YTNWYLETLLRQVSNGHIAYFPAMKAFVNLPTENELTFNAEEYSDISEMRSLSELLGPYGMKFLSESLMWHISSQVAELKKLVVENV<br>DVLTQMRTSFDKPDQMAALFKRLSSVDSVLKRMTIIGVILSFRSLAQEALRDVLSYHIPFLVSSIEDFKDHIPRETDMKVAMNVYELS<br>SAAGLPCEIDPALVVALSSQKSENISPEEEYKIACLLMVFVAVSLPTLASNVMSQYSPAIEGHCNNIHCLAKAINQIAAALFTIHKGSIE<br>DRLKEFLALASSSLLKIGQETDKTTTRNRESVYLLLDMIVQESPFLTMDLLESCFPYVLLRNAYHAVYKQSVTSSA

Supplementary Table 2. Sequences of recombinant proteins used in this study. Note that only sequences in the final product (i.e., after protease cleavage to remove the affinity tag) are shown and are annotated by corresponding colors.
